# A lncRNA landscape in breast cancer reveals a potential role for AC009283.1 in proliferation and apoptosis in HER2-enriched subtype

**DOI:** 10.1038/s41598-020-69905-z

**Published:** 2020-08-04

**Authors:** Alberto Cedro-Tanda, Magdalena Ríos-Romero, Sandra Romero-Córdoba, Mireya Cisneros-Villanueva, Rosa Gloria Rebollar-Vega, Luis Alberto Alfaro-Ruiz, Silvia Jiménez-Morales, Carlos Domínguez-Reyes, Felipe Villegas-Carlos, Alberto Tenorio-Torres, Veronica Bautista-Piña, Fredy Omar Beltrán-Anaya, Alfredo Hidalgo-Miranda

**Affiliations:** 1 Laboratorio de Genómica del Cáncer, Instituto Nacional de Medicina Genómica, Mexico City, Mexico; 2 FUCAM, Instituto de Enfermedades de la Mama, Mexico City, Mexico; 3 Programa de Doctorado en Ciencias Biomédicas, Facultad de Medicina, Universidad Nacional Autónoma de México (UNAM), Mexico City, Mexico; 4 Genomics Laboratory, Red de Apoyo a la Investigación, Universidad Nacional Autónoma de México, Instituto Nacional de Ciencias Médicas y Nutrición Salvador Zubirán, Mexico City, Mexico; 5 Biochemistry Department, Instituto Nacional de Ciencias Médicas y Nutrición Salvador Zubirán, Mexico City, Mexico; 6 Programa de Doctorado de Ciencias Biológicas (Biomedicina), Universidad Nacional Autónoma de Mexico, Ciudad de México, Mexico

**Keywords:** Breast cancer, Long non-coding RNAs

## Abstract

Breast cancer is the most commonly diagnosed neoplasm in women worldwide with a well-recognized heterogeneous pathology, classified into four molecular subtypes: Luminal A, Luminal B, HER2-enriched and Basal-like, each one with different biological and clinical characteristics. Long non-coding RNAs (lncRNAs) represent 33% of the human transcriptome and play critical roles in breast carcinogenesis, but most of their functions are still unknown. Therefore, cancer research could benefit from continued exploration into the biology of lncRNAs in this neoplasm. We characterized lncRNA expression portraits in 74 breast tumors belonging to the four molecular subtypes using transcriptome microarrays. To infer the biological role of the deregulated lncRNAs in the molecular subtypes, we performed co-expression analysis of lncRNA–mRNA and gene ontology analysis. We identified 307 deregulated lncRNAs in tumor compared to normal tissue and 354 deregulated lncRNAs among the different molecular subtypes. Through co-expression analysis between lncRNAs and protein-coding genes, along with gene enrichment analysis, we inferred the potential function of the most deregulated lncRNAs in each molecular subtype, and independently validated our results taking advantage of TCGA data. Overexpression of the AC009283.1 was observed in the HER2-enriched subtype and it is localized in an amplification zone at chromosome 17q12, suggesting it to be a potential tumorigenic lncRNA. The functional role of lncRNA AC009283.1 was examined through loss of function assays in vitro and determining its impact on global gene expression. These studies revealed that AC009283.1 regulates genes involved in proliferation, cell cycle and apoptosis in a HER2 cellular model. We further confirmed these findings through ssGSEA and CEMITool analysis in an independent HER2-amplified breast cancer cohort. Our findings suggest a wide range of biological functions for lncRNAs in each breast cancer molecular subtype and provide a basis for their biological and functional study, as was conducted for AC009283.1, showing it to be a potential regulator of proliferation and apoptosis in the HER2-enriched subtype.

## Introduction

Breast cancer (BRCA) is one of the most common tumors in women around the world. Incidence of this disease is increasing, particularly in countries with emerging economies. In Mexico, BRCA affects about 27,830 women and represents the first cause of cancer related deaths, with approximately 6,884 deaths per year^1^. BRCA is a complex and heterogeneous disease. In the clinical setting, breast tumors are classified into clinically relevant groups based on the expression of three main immunohistochemical (IHC) markers: estrogen and progesterone receptors and (Human Epidermal Growth Factor Receptor 2) HER2,triple negative tumors are defined by the absence of the expression of these markers^2^.

Breast tumors can be further subdivided based on messenger RNA (mRNA) expression signatures. One of these, the intrinsic breast tumor signature, identifies sub-groups based on the expression of 50 genes (the PAM50 signature): Luminal A, Luminal B, HER2-enriched, and Basal-like tumors^3,4^. More importantly, each of these tumor-subtypes has been associated with particular biological and clinical behaviors. Compared to luminal subtypes, Basal-like and HER2-enriched subtypes present worse five-year prognosis, and the HER2-enriched subtype has the worst relapse-free survival^5^. Several lines of evidence indicate that the most representative breast tumor subtype phenotypes identified by mRNA intrinsic signatures reflect not only the alteration of mRNA expression levels, but also the genetic and epigenetic alterations that contribute to the establishment and maintenance of different cancer pathways^6^.

Most of the transcriptional differences between breast tumor subtypes focus on mRNA expression analysis. However, coding transcripts only represent around 39% of the human transcriptome, the rest are non-coding RNAs, which can be divided into two classes: small non-coding RNAs (less than 200 nucleotides, this category includes microRNAs) and long non-coding RNAs (lncRNAs), with more than 200 nucleotides^7^.

LncRNAs have several important biological roles, regulating gene expression at the epigenetic, transcriptional and post-transcriptional levels, and their deregulation has been associated with cancer^8^. In BRCA, lncRNAs are emerging as master regulators in tumor biology, and have oncogenic and tumor suppressor functions related to initiation and cancer progression, some examples are HOTAIR, MALAT-1, lincRNAp21, and GAS5. Deregulation of these lncRNAs is associated with biological functions such as invasion, proliferation, apoptosis, cell cycle, and clinical features such as survival, progression and risk of metastasis^9,10^.

Analyses of lncRNA expression patterns in BRCA have identified differences between tumor groups defined by IHC markers, including HER2 positive^11^ and triple-negative tumors^12^. Other studies have evaluated the aberrant expression of lncRNAs in gene expression-based tumor subtypes, identifying specific lncRNA expression patterns for each subtype^13,14^. Nevertheless, there is still little information about the biological roles of specific differentially expressed lncRNAs in each tumor molecular subtype, particularly in those with worse prognosis, such as HER2-enriched.

The aim of the present study was to explore the transcriptional lncRNA landscape across the four molecular subtypes in 74 Mexican breast tumors. We further validated altered lncRNA expression in the Cancer Genome Atlas (TCGA) cohort. We identified aberrantly expressed lncRNAs in each BRCA molecular subtype, and then selected a group of lncRNAs with exclusive deregulation in each subtype and inferred their potential biological relevance through in silico analysis. Focusing on the HER2-enriched subtype, which has the worst relapse-free and overall survival rates, we identified AC009283.1 as the most up-regulated lncRNA, with a previously unknown biological role. Knockdown of AC009283.1 showed reduced cell proliferation, promoted S phase arrest, and enhanced apoptosis in SKBR3 cells (HER2-enriched). These biological processes were also enriched in HER2-enriched breast cancer tumors with high AC009283.1 expression levels, suggesting that AC009283.1 plays a role in promoting HER2-enriched tumors. The influence of lncRNAs on deregulated pathways in different breast tumor subtypes is relevant and supports the need for new approaches to understand the biology of BRCA.

## Results

### lncRNA expression is deregulated in breast cancer

**Figure 1:**
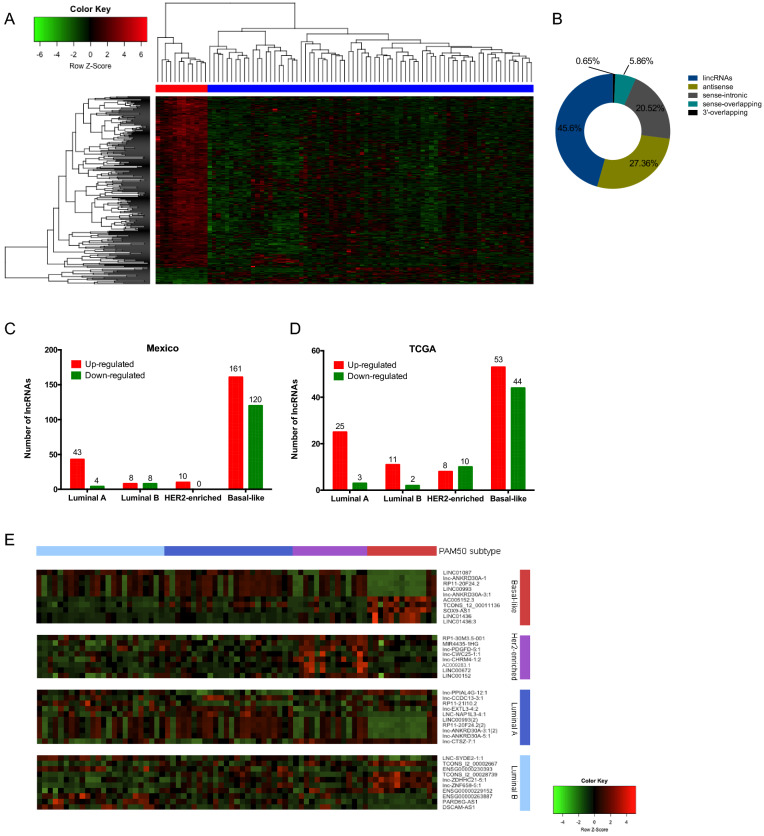
(**A**) Supervised hierarchical cluster analysis of the expression of the 307 lncRNAs deregulated in breast tumor (blue) and normal tissue (red). (**B**) Classification of lncRNAs deregulated between tumor and adjacent tissues, grouped based on their genomic localization by intersection with protein-coding genes. (**C**) lncRNAs deregulated across molecular subtypes in the Mexican cohort (fold change > 1.5, < − 1.5, FDR < 0.05) and (**D**) in the TCGA project (Fold change log 1.0. adj. p-value < 0.05). (**E**) Top ten lncRNAs deregulated across molecular subtypes in the Mexican cohort. Basal-like in red, Her2-enriched in purple, Luminal A in blue and Luminal B in cyan. Each column represents a sample and each row a lncRNA.

The transcriptional portrait among tumors and adjacent tissues revealed 307 differentially expressed lncRNAs (27 up-regulated and 280 down-regulated) (fold change > 2.0 and < − 2.0 and FDR < 0.05) (Supplementary data 1.1). Hierarchical clustering analysis based on differentially expressed lncRNAs was able to optimally discriminate tumor breast tissues from normal adjacent samples, as shown in the heat map (Fig. 1A). The altered lncRNAs were classified into ncRNA categories as follows: lincRNAs (45.6%), antisense (27.36%), sense-intronic (20.52%), sense-overlapping (5.86%) and 3′-overlapping (0.65%) (Fig. 1B).

Several differentially expressed lncRNAs identified in our study have been previously reported to be related to BRCA, such as HOTAIR, HOTAIRM1, XIST, PANDAR, EPB41L4A-AS1, BC040587, FGF14-AS2, DSCAM-AS1, LINC00472, MIR31HG and FAM83H-AS1 (Supplementary data 1.2).

A set of differentially expressed lncRNAs was independently validated using whole transcriptome RNA-seq analysis data from TCGA. Some of the lncRNAs described as altered in our profile did not significantly change in the TCGA dataset while others were not detected by the sequencing experiment (Supplementary data 1.3).

### Molecular subtypes are associated with distinct clinical outcomes

A total of 74 breast tumor samples and 12 adjacent tissue samples were included in the PAM50 intrinsic subtype analysis. Incidence of PAM50 subtypes were reported as follows: 32.4% Luminal A (n = 24), 31.1% Luminal B (n = 23), 18.9% HER2-enriched (n = 14), and 17.6% Basal-like (n = 13) subtypes. Clinical behavior differs among the molecular subtypes. For instance, in our in-home profiled cohort, the HER2-enriched subtype showed the highest rate of metastasis (log-rank p = 0.034). Similarly, lower overall-survival was observed in the METABRIC data set (n = 1,463) (95% CI [1.417–2.368] Cox p-value = 2.08E−10) as well as in the TCGA data set (n = 743) (95% CI [0.97–4.01] Cox p-value = 2.19E−7) (Supplementary Figure 1). Clinical and pathological features of our cohort are summarized in Supplementary Table 1.

### Expression profile of lncRNAs in breast cancer subtypes

To understand the biology of the intrinsic breast cancer subtypes, we explored the transcriptional landscape of the lncRNAs along the PAM50 subtypes. 354 differentially expressed lncRNAs were identified. In Luminal A tumors, we observed 47 altered lncRNAs, 16 in Luminal B, 10 in HER2-enriched, and 281 in Basal-like (Fig. 1C) (Supplementary data 2.1). TCGA data showed a similar proportion of lncRNAs deregulated in each molecular subtype (Fig. 1D) (Supplementary data 2.2). Figure 1E shows the expression of the top deregulated lncRNAs (5 up-regulated and 5 down-regulated) in Basal, Luminal A, Luminal B, and eight up-regulated in HER2-enriched tumors in our cohort.

### lncRNA expression portraits in Luminal A tumors

**Figure 2:**
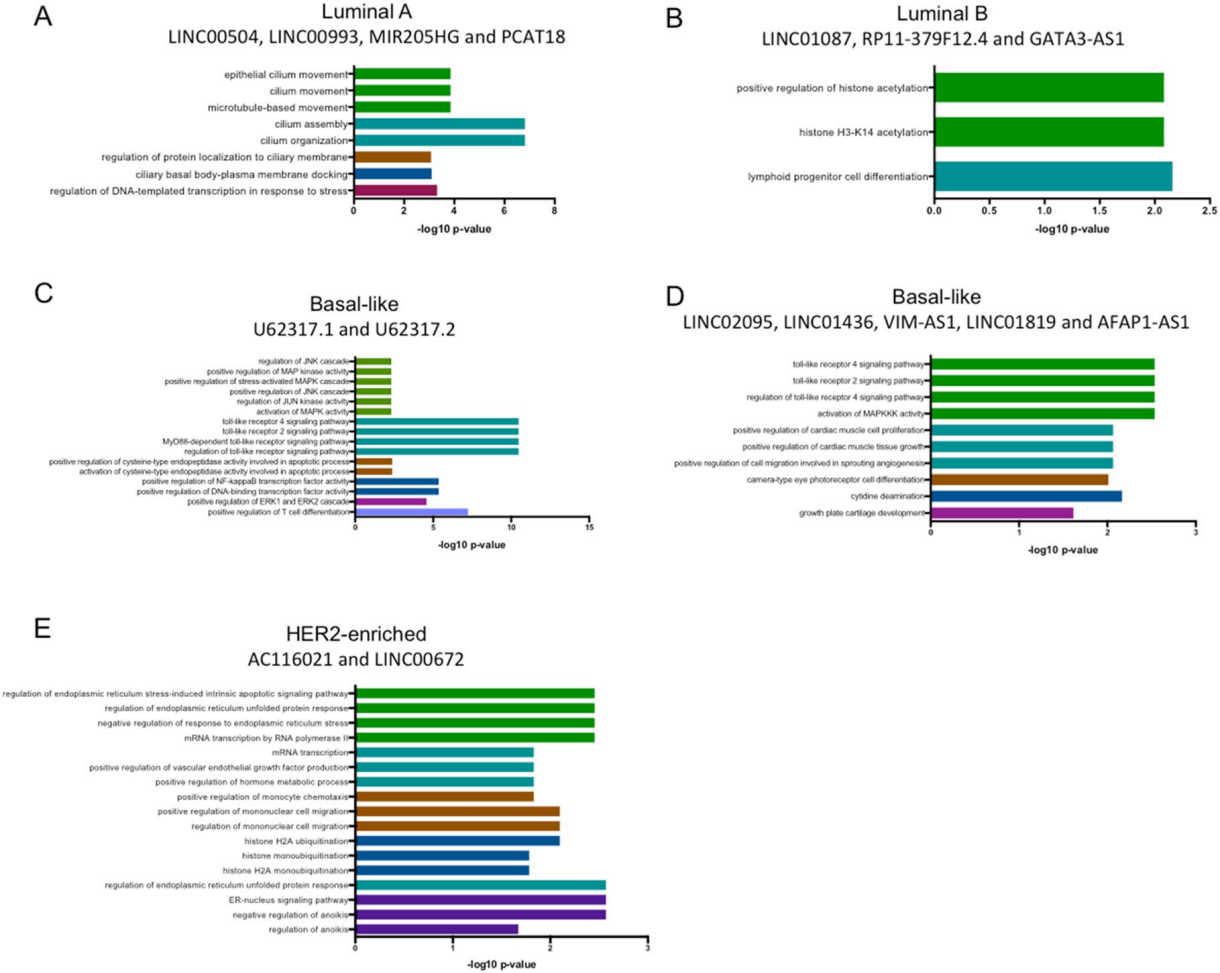
Gene ontology (GO) analysis of lncRNAs up-regulated in our cohort and TCGA. GO was performed using mRNAs that correlate with lncRNAs in each subtype using ClueGo in Cytoscape with PValue Corrected < 0.05.

In our cohort, we identified 47 lncRNAs differentially-expressed in the Luminal A subtype (43 up-regulated and 4 down-regulated) (Supplementary data 2.1). To define robust altered lncRNAs, we examined the TCGA cohort. Four lncRNAs were commonly up-regulated in Mexican and TCGA cohorts: LINC00504, LINC00993, MIR205HG and PCAT18. To get insight into their biological role as transcriptional regulators we investigated co-expression lncRNA–mRNA patterns of these four lncRNAs. 340 mRNAs were significantly correlated with LINC00504, LINC00993, MIR205HG and PCAT18 in both cohorts (R > 0.3 p-value < 0.05) (Supplementary data 3.1). Gene ontology (GO) analysis of the correlated mRNAs identified positive regulation of DNA-templated transcription in response to stress, microtubule-based movement and processes related to ciliary docking and organization (Fig. 2A).

### lncRNA expression portraits in Luminal B tumors

We identified 16 deregulated lncRNAs in the Luminal B subtype in our cohort (8 up-regulated and 8 down-regulated) (Supplementary data 2.1). Up-regulation of LINC01087, RP11-379F12.4 and GATA3-AS1 was validated in the TCGA data set. Correlation analysis of the altered lncRNA and mRNAs, detected 70 mRNAs co-expressed in both cohorts (Supplementary data 3.2). According to GO analysis, the correlated genes participate in the regulation of histone H3-K14 acetylation and lymphoid progenitor cell differentiation (Fig. 2B). Moreover, in the TCGA cohort, the over-expression of these lncRNAs (LINC01087, RP11-379F12.4) was significantly associated with increased survival rates (log-rank test < 0.05), compared with tumors with low expression levels (Supplementary figure 2).

### lncRNA expression portraits in basal-like tumors

The basal-like subtype showed the highest number of differentially expressed lncRNAs, with 161 up-regulated and 120 down-regulated transcripts in our cohort (Supplementary data 2.1). Regarding the up-regulated lncRNAs, only a low number of them have been previously implicated in cancer, such as the up-regulated CASC15, LINC00662, LINC01272, LINC00857, LINC00092, LINC00673 and the down-regulated LINC00657, LINC01087 and LINC00993. LncRNAs U62317.1 and U62317.2 are adjacent lncRNAs localized in chromosome 22q13.33, and both are up-regulated in our samples and in the TCGA cohort. U62317.1 and U62317.2 expression was correlated with 419 mRNAs in both cohorts (Supplementary data 3.3). GO analysis showed that these mRNAs are implicated in positive regulation of JNK cascade, activation of cysteine-type endopeptidase involved in apoptotic process, positive regulation of NF-kappaB, ERK1 (MAPK3) and ERK2 (MAPK1), and positive regulation of T cell differentiation (Fig. 2C). High expression of U62317.2 was associated with higher OS in patients with basal subtype (log-rank p-value = 0.04378) (Supplementary Figure 3). Moreover, another five lncRNAs were common between our cohort and TCGA: LINC02095, LINC01436, VIM-AS1, LINC01819 and AFAP1-AS1. Co-expression analysis revealed 103 correlated mRNAs in both cohorts (Supplementary data 3.3), which are associated with the activation of MAPKKK and positive regulation of cell proliferation and differentiation (Fig. 2D).

**Figure 3:**
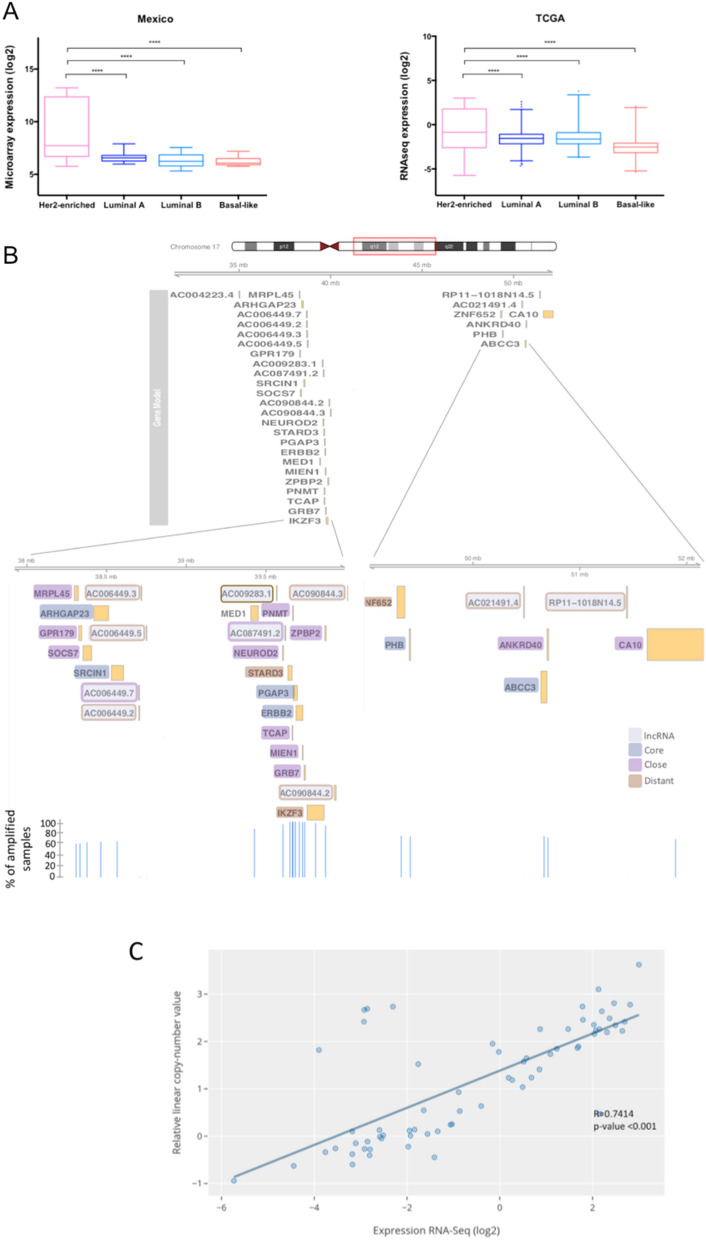
(**A**) AC009283.1 expression across different molecular subtypes in the Mexican cohort (n = 74; microarray data) and TCGA/TANRIC cohort (n = 853; RNA-Seq data). (**B**) Map of amplicon 17q12 with mRNAs and lncRNAs. (**C**) Correlation analysis between expression and copy-number value of AC009283.1 in HER2-enriched from TCGA data.

### lncRNA expression portraits in HER2-enriched tumors

We detected 8 novel up-regulated lncRNAs in the HER2-enriched subtype, not reported previously in BRCA (Supplementary data 2.1). Two of them (AC116021 y LINC00672) are up-regulated in both cohorts (our study and TCGA), and their expression is correlated with 90 mRNAs in both cohorts (Supplementary data 3.4). Gene ontology analysis revealed their involvement in the positive regulation of RNA Pol II transcription, response to endoplasmic reticulum stress, positive regulation of VEGF production, positive regulation of monocyte chemotaxis, histone monoubiquitination, negative regulation of anoikis, among other processes (Fig. 2E).

As described above, HER2-enriched breast cancer patients showed a significantly decreased global survival rate, both in TCGA and METABRIC cohorts. Therefore, we endeavored to characterize altered lncRNAs in this molecular subtype. Interestingly, AC009283.1 presented the highest expression level in HER2-enriched tumors compared with other BRCA intrinsic molecular subtypes, in both analyzed datasets (Mexican cohort: FC 4.02, p value < 0.0001, TCGA: FC 2.46 and p-value < 0.0001) (Fig. 3A), without any previous functional report (Supplementary data 2.1). Therefore, AC009283.1 was selected for further in vitro evaluation.

### AC009283.1 is up-regulated and amplified in HER2-enriched tumors

AC009283.1 is an intergenic lncRNA localized in chr17:39,566,915–39,567,559 (GRCh38/hg38) annotated in the ENSEMBL database as ENSG00000273576. Some alternative names have been reported in other databases such as HSALNG0116248, NONHSAG021701, lnc-CDK12-1 and RP11-390P24.1**.** The Coding potential (CP) of AC009283.1 is 0.113, according to the Coding Potential Calculator, similar to other well-known lncRNAs, such as HOTAIR, MALAT-1 and PANDAR (CPs: 0.09, 0.1295 and 0.058, respectively).

AC009283.1 is significantly up-regulated in the HER2-enriched subtype in our Mexican cohort, with a fold change of 4.02 (microarray analysis; p-value < 0.0001) (Supplementary data 2.1). Independent validation with TCGA data showed an increased expression in HER2 enriched tumors (RNA-Seq analysis; foldchange 2.46 and p-value < 0.0001) (Fig. 3A). Breast cancer genomic analysis revealed that AC009283.1 maps close to the HER2 gene and is frequently co-amplified within the core region of the HER2-amplicon (17q12) (Fig. 3B). Dedicated copy number alteration analysis on TCGA HER2-amplified tumors have demonstrated that the amplicon can span over other neighboring regions on 12q-21.3, where a number of protein-coding and non-coding genes are encoded and consequently amplified (Fig. 3B). The expression levels of AC009283.1 were significantly correlated with HER2 copy number alterations evaluated through the Genomic Identification of Significant Targets in Cancer (GISTIC) from TCGA data (Fig. 3C). Overall, these data suggest that AC009283.1 is a relevant feature in HER2-enriched breast cancer programs. Thus, to investigate whether the altered expression of AC009283.1 affects HER2-enriched tumor biology, we studied its in-vitro activity in a HER2-enriched cell line model.

Expression of AC009283.1 was evaluated in a panel of BRCA cell lines, identifying SKBR3 cell line as a potential biological model for further analysis. SKBR3 cells over-expressing AC009283.1, carry a 17q12 amplification, and according to PAM50 subtypes represents a HER2-enriched subtype (Supplementary Figure 4). To infer the regulatory role of the lncRNA in cancer programs, its subcellular localization was evaluated. Overall, AC009283.1 was preferentially located within the nucleus, which may suggest its predominant function as a regulator of protein-coding gene expression through transcriptional and epigenetic mechanisms (Supplementary Figure 5).

### Knockdown of AC009283.1 alters the transcriptional profile of HER2-enriched breast cancer cells

**Figure 4:**
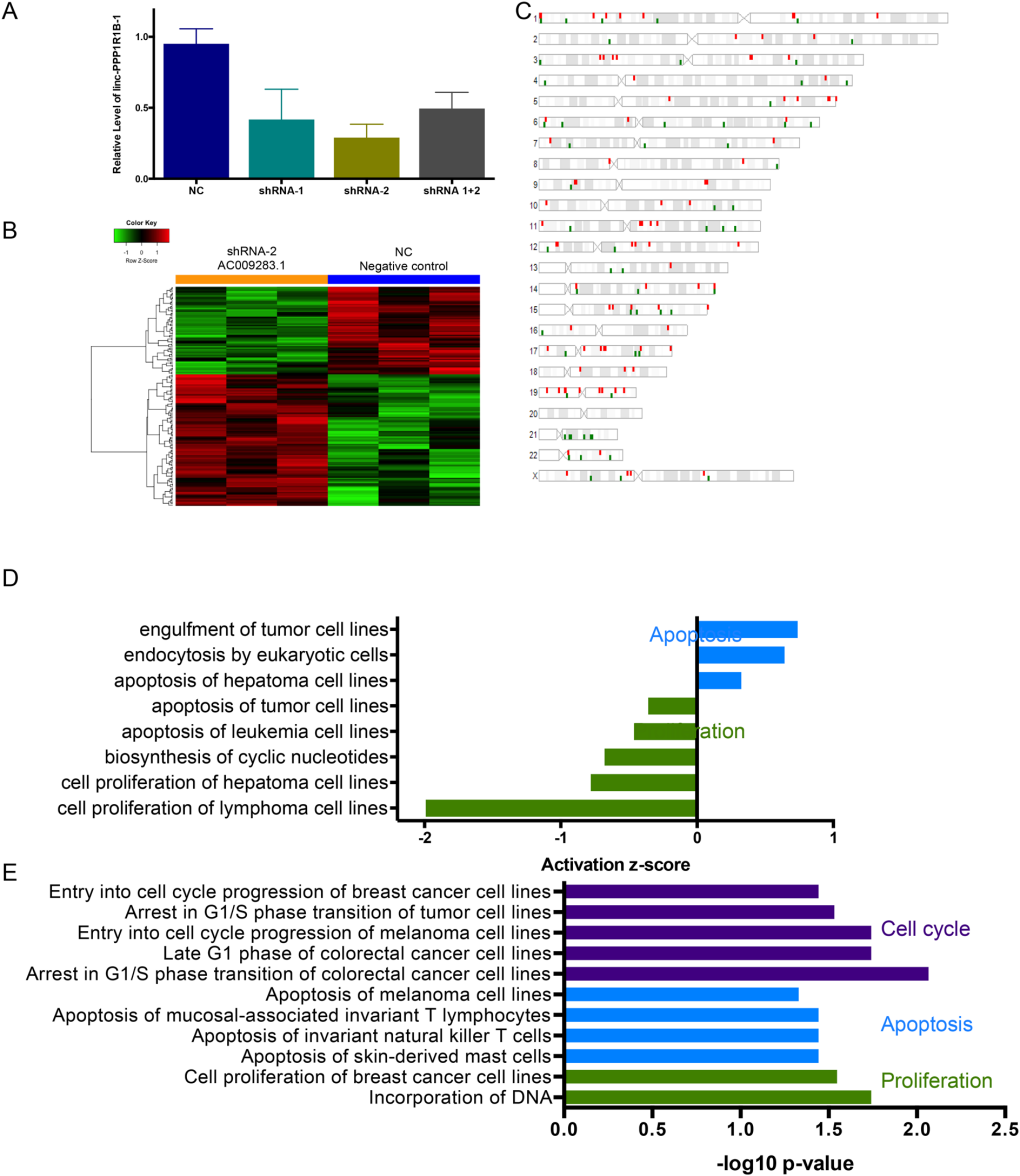
(**A**) Real-time qPCR illustrating gene silencing by shRNA against AC009283.1 in SKBR3, a HER2-enriched cell line model. (**B**) Supervised hierarchical cluster analysis shows 158 differentially expressed genes after AC009283.1 knockdown (Foldchange < − 1.2, > 1.2 p-value < 0.05); we observed 94 up-regulated and 64 down-regulated genes in shRNA-2 vs NC and (**C**). Their distribution across the genome. (**D**) Ingenuity Pathway Analysis of 158 genes differentially expressed after knockdown of AC009283.1 in SKBR3 cells. (**E**) Enrichment pathway analysis of genes differently expressed in samples of HER2-enriched tumors from TCGA with high vs low expression of AC009283.1.

To explore AC009283.1 function in the HER2-enriched BRCA subtype, we inhibited its expression using two shRNA sequences (shRNA1 and shRNA2). Both sequences target a common exon in 10 of 14 isoforms reported for AC009283.1. 96 h post-transfection, the most relevant reduction of AC009283.1 expression level (70% *vs* negative control) was achieved with shRNA2, thus this shRNA sequence was used for further experiments (Fig. 4A).

Since lncRNAs are capable of regulating genes located nearby (*cis*) or in distant genomic regions (*trans*), we used a high-throughput microarray approach to evaluate transcriptome alterations after AC009283.1 shRNA-mediated knockdown, comparing with shRNA negative control, in SKBR3 cells. 158 genes were differentially expressed among the experimental conditions and distributed throughout the genome (> 1.2, < − 1.2-fold) (ANOVA p-value < 0.05) (Fig. 4B,C). Genes such as *CASCA4*, *NOCTH3*, *TNFa*, *FOSB*, *BLC2A1*, *DNML1,* and *KLF6* were deregulated in AC009283.1 knockdown cells (Supplementary data 4.1). To further to explore the effect of inhibiting AC009283.1 in biological processes, we performed a pathway enrichment analysis of the differentially expressed genes, showing proliferation and apoptosis as significantly affected pathways (Fig. 4D). Other oncogenic pathways were also altered, such as oxidative phosphorylation, mitochondrial dysfunction, p38 MAPK Signaling, PI3K/AKT signaling, among others (Supplementary Figure 5). Interestingly, proliferation, apoptosis and cell cycle were also observed in HER2-enriched tumors from patients in TCGA containing up-regulated AC009283.1 (upper vs lower quartile) (Fig. 4E) (Supplementary data 4.2). These results suggest that AC009283.1 plays a role in biological processes by regulating gene expression of important genes related with carcinogenic pathways.

### AC009283.1 knockdown inhibits proliferation and induces apoptosis in HER2-enriched breast cancer cells

**Figure 5:**
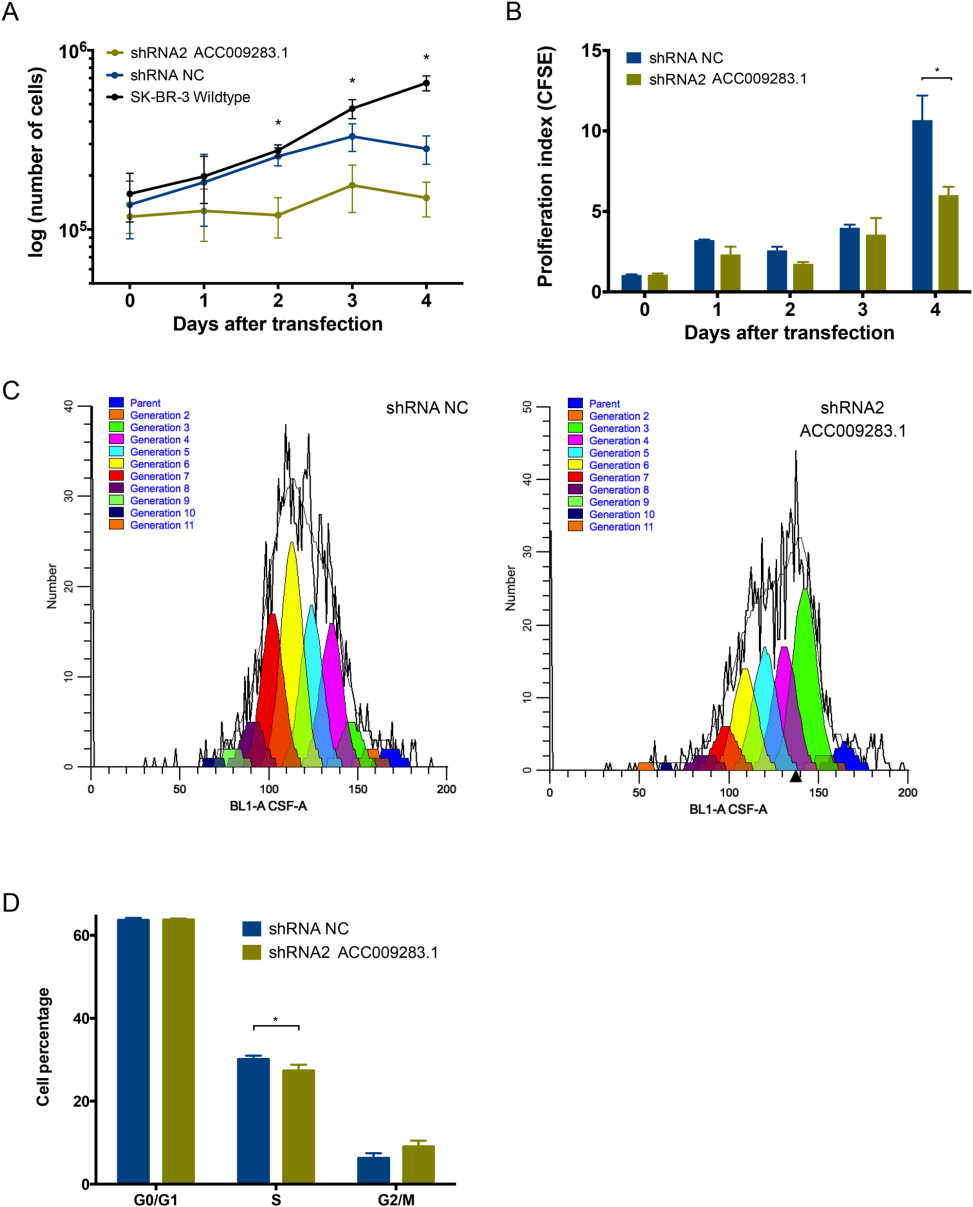
(**A**) Proliferation assay performed following knockdown of AC009283.1 with shRNA2, shRNA NC as negative control, and wildtype cell line. Cells were counted every day for four days. *p < 0.05, **p < 0.005, ***p < 0.0005, comparing with shRNA NC for each condition via Student’s *t* test. (**B**) Proliferation index using CFSE assay in SKBR3 cells with shRNA2 AC009283.1 and shRNA NC. *p < 0.05, **p < 0.005, ***p < 0.0005, comparing with shRNA NC for each condition via Student’s *t* test. (**C**) Representative CFSE flow-cytometry histograms showing the effect on in vitro SKBR-3 cell proliferation with shRNA NC and shRNA2 (AC009283.1 knock-down). (**D**) Cell cycle assay with flow-cytometry using (propidium iodide) PI on in vitro SKBR-3 cells, AC009283.1 knockdown causes accumulation of cells at S phase. *p < 0.05, **p < 0.005, ***p < 0.0005, comparing to shRNA NC for each condition via Student’s *t* test.

To validate the pathway analysis, we performed cell proliferation and apoptosis assays in vitro. Automated cell counting showed that knockdown of AC009283.1 attenuated cell proliferation compared with the control group (NC) in the SKBR3 cell line. Significantly lower cell numbers are observed from the second day after transfection (Fig. 5A). To confirm this result, proliferation rates were evaluated by flow cytometry. The proliferation index on the fourth day after transfection was significantly decreased (6.01 vs 10.68 in negative control cells) in SKBR3 cells with reduced AC009283.1 (Fig. 5B). A representative graph of the decreased proliferation rates observed with CFSE assay in AC009283.1 knockdown cells is represented in Fig. 5C. To understand how cell proliferation is controlled by AC009283.1, we performed a cell cycle assay after four days post-transfection with shRNA2 against AC009283.1. A significant arrest in S phase was observed in cells with low levels of AC009283.1 (Fig. 5D). These results suggest that AC009283.1 may regulate cell proliferation by arresting cells in S phase.

**Figure 6:**
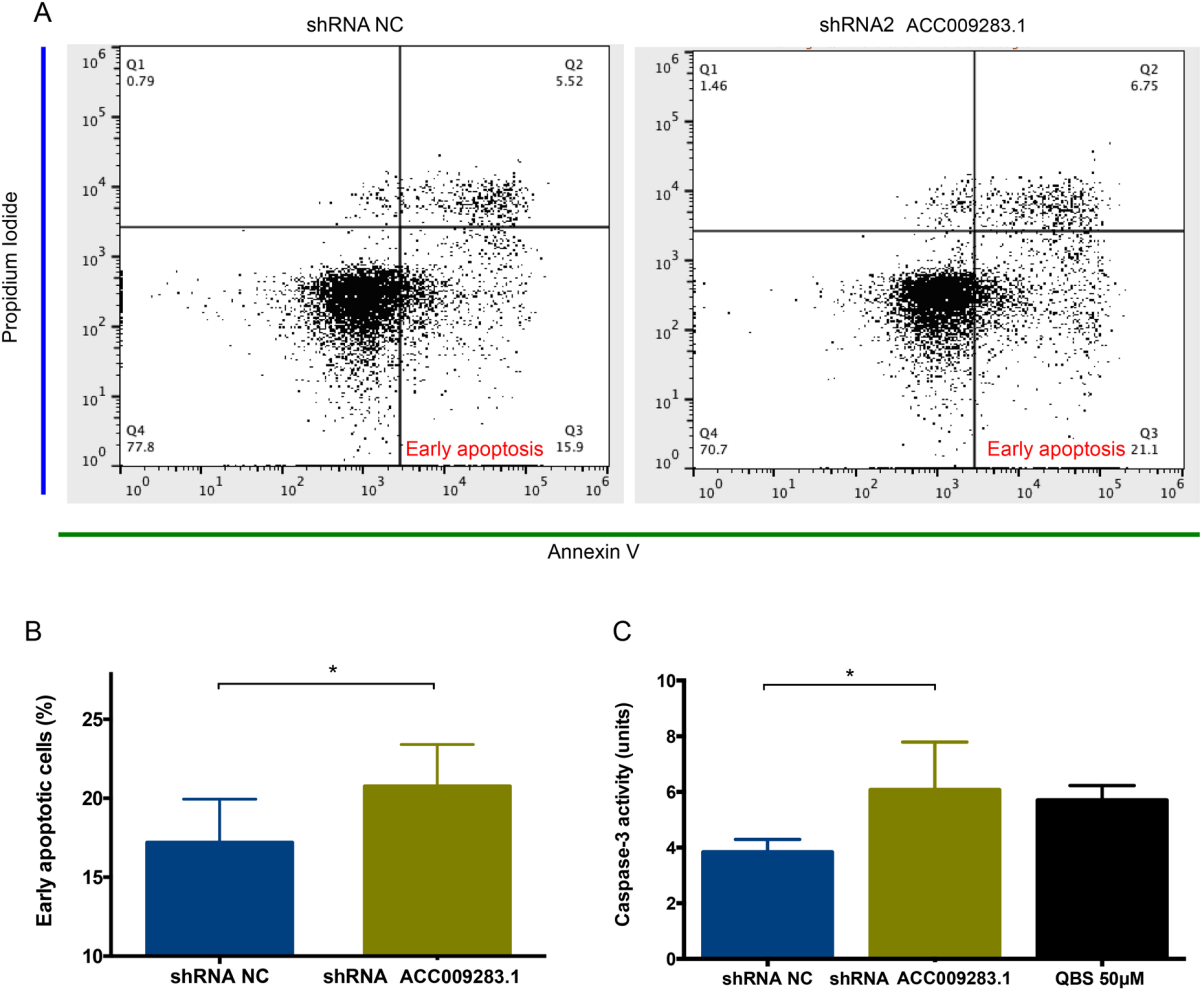
(**A**) Early apoptosis cells were observed to be significantly different between shRNA NC and shRNA2 AC009283.1 using flow cytometry assay on SKBR-3 cells. (**B**) Analysis that represents the percentage of cells in early apoptosis in three biological assays on SKBR-3 cells. *p < 0.05, **p < 0.005, ***p < 0.0005, compared with shRNA NC for each condition via Student’s *t* test. (**C**) Quantitative analysis of caspase-3 activity of cells treated with shRNA NC and shRNA2 AC009283.1, units are defined as the amount of enzyme that cleaves 1 nm colorimetric substrate/h. QBS 50uM was used as the positive control for apoptosis. *p < 0.05, **p < 0.005, ***p < 0.0005, compared with shRNA NC for each condition using Student’s *t* test.

Additionally, apoptosis assays using flow cytometry revealed that AC009283.1 knockdown enhances the percentage of cells undergoing early apoptosis (21.3% vs 15.9% in control cells) (Fig. 6A,B). Furthermore, we measured caspase-3 enzymatic activity and found that AC009283.1 knockdown significantly increases the level of caspase-3 activity at day four, compared with negative control cells (shRNA NC) (Fig. 6C). These results suggest that AC009283.1 promotes apoptosis resistance in HER2-enriched breast cancer cells.

### AC009283.1 is over-expressed in HER2-amplified tumors and might regulate relevant oncogenic pathways

Based on the above described correlation between HER2-gain and the lncRNA level, we hypothesized that AC009283.1 over-expression is not unique to the HER2-enriched subtype but rather an event associated with HER2-amplification. Since the HER2-enriched PAM50 subtype captures some, but not all of the HER2+ tumors, we expanded our analysis to all HER2-amplified tumors, evaluated through microarray or immunochemistry (IHC) analysis, from public available datasets from TCGA and Gene Expression Omnibus (GEO).

**Figure 7:**
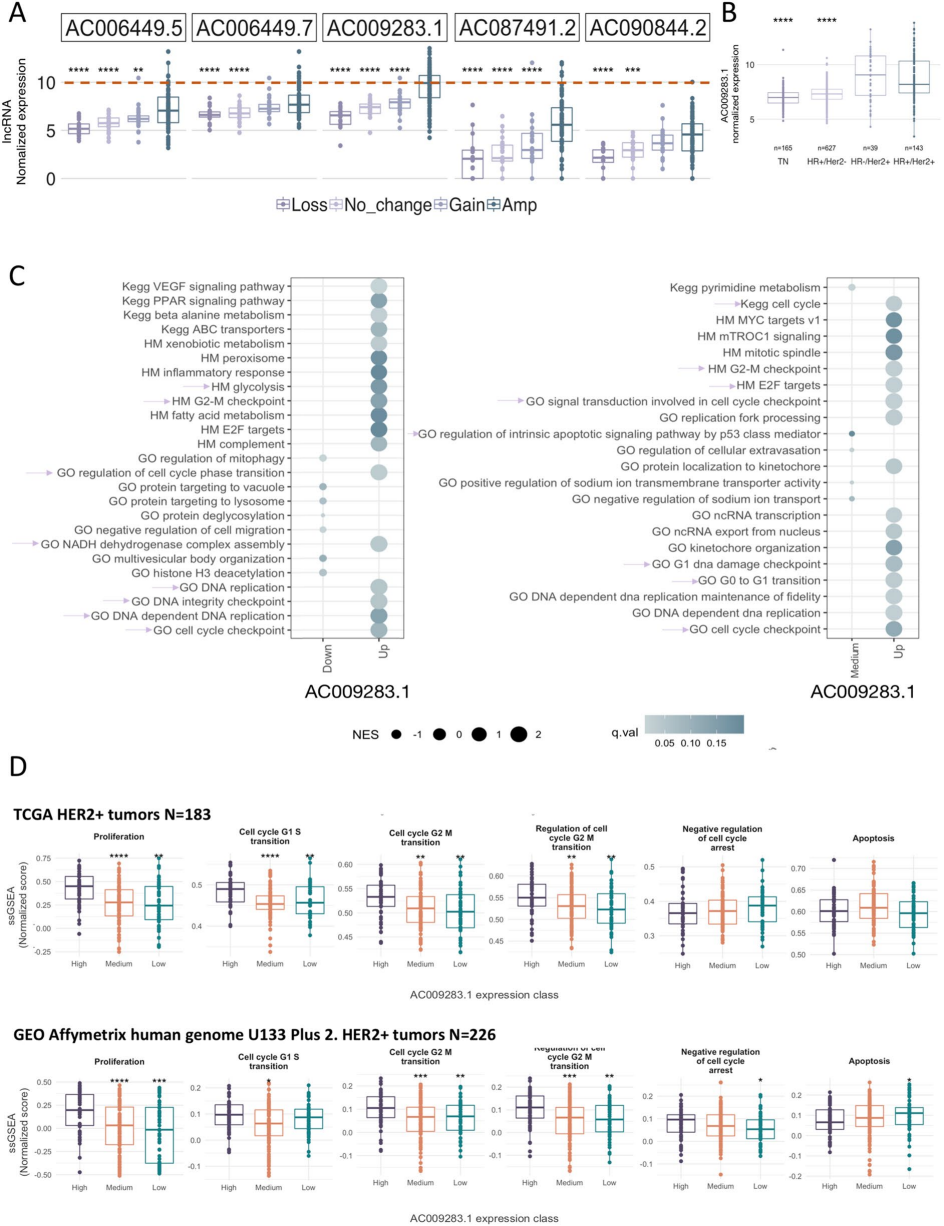
(**A**) Top 5 lncRNAs expressed in 17q12-21.3 vs copy number changes. (**B**) Expression of AC009283.1 in BRCA subtypes including hormone receptor and HER2 receptor status. (**C**) Bubble plot representing GSEA analysis in AC009283.1 high expression breast cancer samples versus AC009283.1 medium and low expression samples. Pink arrows depict biological processes previously shown in in vitro shRNA-mediated knockdown assays. (**D**) ssGSEA analysis for AC009283.1 high, medium and low expression levels, depicting enrichment for cellular proliferation, cell cycle and apoptosis processes in an independent HER2-enriched breast cancer cohort.

We first explored the expression portraits of HER2-neighboring lncRNA genes encoded in the 17q12-21.3 amplicon. We systematically explored and integrated somatic copy number alteration data of HER2 amplification in breast tumors, independently of the PAM50 molecular subtype (Fig. 3B). A set of lncRNAs contained in or neighboring amplified genes within the 17q12-21.3 amplicon showed a coordinated expression pattern with HER2 mRNA level, suggesting that these correlated transcripts may be needed to sustain oncogenic programs prompted by HER-amplification (Supplementary Figure 7). Importantly, AC009283.1 was among the lncRNAs most correlated with ERBB2 expression (R:0.61 p < 0.001) (Supplementary Figure 7). Furthermore, among the overall evaluated lncRNAs, AC009283.1 presented the highest expression in HER-amplified tumors compared with tumors without copy number changes or amplicon loss (Fig. 7A). In accordance with these data, we found a significant up-modulation of AC009283.1 in HER2+ tumors evaluated by IHC, independent of the hormone receptor status (Fig. 7B).

After determining a relevant correlation between HER2-amplification/over-expression and AC009283.1 expression, we performed a gene set enrichment analysis (GSEA) to identify relevant biological processes enriched in HER2+ tumors with diverse AC009283.1 expression patterns. As shown in Fig. 7C, GSEA analysis confirm previous findings in our in vitro assays. We found a significant, exclusive enrichment in G2–M checkpoint, G0–G1 transition, regulation of cell-cycle phase transition, DNA replication, and regulation of intrinsic apoptotic signaling pathways in HER2+ tumors over-expressing AC009283.1.

To validate our observations in a more sophisticated characterization, normalized ssGSEA (single-sample GSEA) scores were computed on HER2+ breast cancer samples retrieved from TCGA (n = 183) and Gene Expression Omnibus (GEO) datasets (n = 226). Once again, tumors over-expressing AC009283.1 (High group) were found to be predominantly enriched in cell proliferation, cell cycle, cell cycle G1–S transition, cell cycle G2–M transition, regulation of cell G2–M transition and negative regulation of cell cycle arrest, compared with low-expressing tumors (Fig. 7D).

### Modular expression analysis of AC009283.1 in HER2-amplified tumors

**Figure 8:**
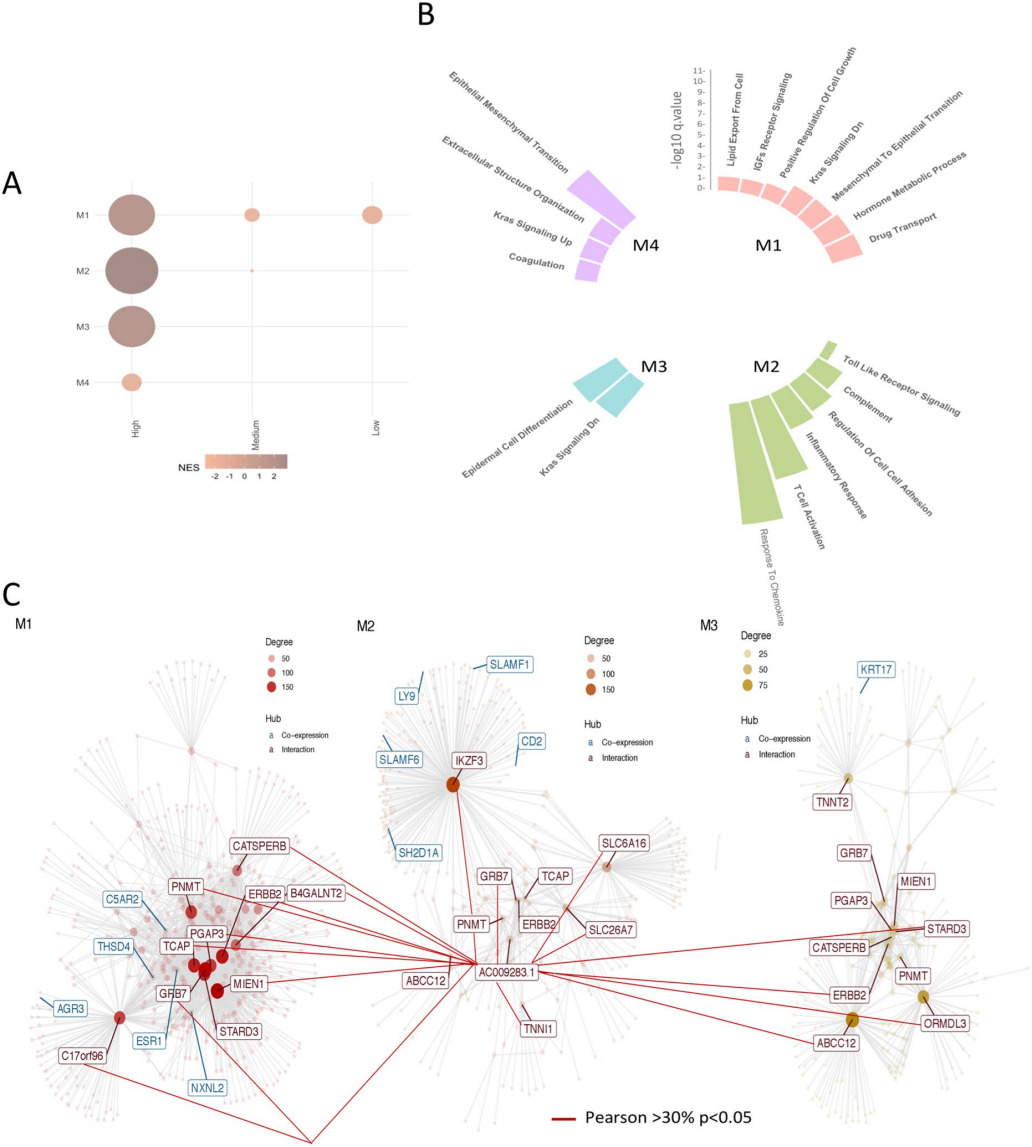
(**A**) Co-expression modules detected by CEMiTool and their enrichment in tumors with high, medium and low expression of AC009283.1. (**B**) Representation of biological processes in each gen module. (**C**) Network graph with the lncRNA in the center and its relationship of co-expression with genes from modules 1, 2 and 3.

Assessing co-expression patterns may help identify novel functional connections between AC009283.1 and mRNAs under HER2+ amplification. Thus, we performed a gene co-expression network analysis with CEMiTool package using the expression profiles of HER2+ tumors divided in accordance with AC009283.1 levels into a low, medium or high category. Four-gene modules were revealed (Fig. 8A). M1, M2 and M3 were highly enriched in AC009283.1 (Fig. 8A) and gene-set enrichment analysis revealed a significant over-representation of cell metabolism and growth (M1), immune signaling (M2) and oncogenic pathways, such as KRAS (M3), of all them markedly related with aberrant cell proliferation, cell-cycle progression and apoptotic rates, which corroborated our in vitro results (Fig. 8B).

We then integrated co-expression information to identify main regulators and hubs within the modules. Interestingly, as shown in the interaction network, AC009283.1 correlated with genes contained in modules M1, M2 and M3 (Fig. 8C). This analysis showed that AC009283.1 lncRNA may unveil potential transregulatory relationships within the HER2 response network.

Overall, these data suggested that high expression of AC009283.1 is strongly associated with cell proliferation, cell cycle progression and apoptosis, and further reveal that the biological role of AC009283.1 in HER2-enriched breast cancer is probably modulated and associated with HER2-amplification.

## Discussion

A few decades ago, the non-coding sequences of the genome (98%) were considered non-functional and the search for tumor oncogenes and suppressors focused only on protein coding sequences (2%). Now with the use of high-throughput screening we know that around 70% of the genome is transcribed, including non-coding regions which play an important role in the regulation of gene expression^15^. LncRNAs are deregulated in several cancer types^8^. This study provides a whole transcriptome in-house profile from well-characterized breast cancer tumors, and identifies the deregulated expression of AC009283.1 in the HER2-enriched subtype. We found 307 lncRNAs differentially expressed in tumors compared to normal adjacent tissue and their expression pattern was able to distinguish BRCA tissue from normal adjacent. A small set of deregulated lncRNAs have already been reported with a functional role in breast carcinogenesis,however, the biological function of the majority is unknown. Surprisingly, when we validated our gene expression profiles using TCGA data, we found an agreement of 33.8% of the results. The concordance levels between our patients and the TCGA cohort could be influenced by three different variables: (1) sample number disparities (74 for the Mexican cohort and 990 for TCGA), (2) the detection technologies used to obtain gene expression data (microarray for Mexican and RNA-seq for TGCA cohort), and (3) intrinsic biological factors of samples, such as normal/tumor purity percentage. However, the overlapped lncRNAs may have a relevant biological role in breast cancer, as they are present in two independent cohorts. In addition, these data suggest that microarrays and RNA-seq may be complementary methods to identify lncRNAs deregulated in BRCA^16,17^.

To acquire insight into the potential biological role of the altered lncRNAs, in our cohort and the TCGA data, among the molecular subtypes, a co-expression analysis of lncRNA–mRNA was performed. It is noteworthy that co-expression analysis has been described as a robust approach for inferring the biological function of lncRNAs^18^.

In the Luminal A subtype LINC00504, LINC00993, MIR205HG and PCAT18 were found up-regulated in the analyzed cohorts. Their biological function has not been reported, but according to co-expression analysis (lncRNA–mRNA) they are associated with positive regulation of DNA-templated transcription, a process that regulates the frequency, rate and extent of RNA polymerase II transcription, a target for cancer therapy^19^. This observation might also be related to the fact that in Luminal A BRCA cell line (MCF7), estradiol is a potent stimulator of RNA polymerase II transcription^20^. These four lncRNAs up-regulated in Luminal A tumors also regulate microtubule-based movement, a process that results in the movement of organelles through polymerization or depolymerization of microtubules, this process is also a promising target for breast anticancer drugs^21^. Another biological process enriched was ciliary docking and organization. It has been reported that cilia in breast tumors are more frequent in more differentiated tumors such as those in the luminal A subtype^22^. In addition, it was observed that the promoter of MIR205HG shows a binding site for the estrogen receptor in the Luminal A cell line MCF7, according to Chromatin Immunoprecipitation Sequencing (ChIP-Seq) assays^23^^,^ and PCAT18 lncRNA was significantly associated with androgen receptor (AR) signaling in prostate cancer^24^. These data suggest a possible association of these lncRNAs in tumor biology and hormone signaling in this subtype.

In the Luminal B subtype, overexpression of DSCAM-AS1, has been previously reported to mediate tumor progression and tamoxifen resistance. This molecular subtype is the most clinically aggressive ER-positive BRCA, and DSCAM-AS1 is a major discriminator of the luminal subtypes in BRCA^25^. Three other lncRNAs over-expressed in our cohort and TCGA data were LINC0187, RP11-379F12.4 and GATA3-AS1) So far, there are no reports of their biological function in cancer. Co-expression analysis (lncRNA–mRNA) associated them with histone H3K14 acetylation (H3K14ac). Acetylation of specific lysine residues of histones plays a key role in regulating gene expression, in fact, H3K14ac is an active histone mark and correlates with the magnitude of gene expression, probably promoting the expression of oncogenes^26^.

In the Basal-like subtype we observed 280 deregulated lncRNAs, importantly, the function of most of them is still unknown. This makes evident the lack of knowledge about the function of the lncRNAs deregulated in the Basal-like tumor phenotype, one of the most aggressive subtypes lacking targeted therapy. For instance, the up-regulation of lncRNAs U62317.1 and U62317.2 (in our cohort and TCGA) is correlated with genes involved in the positive regulation of the JNK cascade, an intracellular protein kinase cascade. Its known that persistent activation of JNKs is involved in cancer development and progression^27^. Another pathway enriched with these two lncRNAs is the activation of cysteine-type endopeptidases involved in the apoptotic process, such as caspases, enzymes known for their role in the initiation and execution of apoptosis^28^. Additionally, these two lncRNAs are associated with positive regulation of NF-κB, deregulated NF-κB pathway leads to the disruption of the balance between cell proliferation and death through the positive regulation of anti-apoptotic proteins^29^.

Another four lncRNAs (LINC02095, LINC01436, VIM-AS1, LINC01819 and AFAP1-AS1) were also up-regulated in Basal-like subtype in our cohort and the TCGA cohort. They have an expression correlation with 103 mRNAs in both cohorts, associated with activation of MAPKKK (MAP3K1), which functions in cell survival, apoptosis, and cell migration in multiple tumor cell types^30^.

In the HER2-enriched subtype, 8 lncRNAs are up-regulated, of which, only the function of LINC00152 is known, since it regulates cell proliferation, promotes cell cycle arrest at G1 phase, triggers late apoptosis, reduces the epithelial mesenchymal transition program, and suppresses cell migration and invasion in gastric cancer^31^. MIR4435-1HG is up-regulated in HER2-enriched tumors and has been associated with reduced OS and disease-free survival in head and neck cancer^32^. It is noteworthy that the functions of LINC00152 and MIR4435-1HG, mentioned above, have been described in gastric and head and neck cancer models, but not breast cancer.

There are no reports of biological function in cancer of lncRNAs AC116021 and LINC00672, that were found co-expressed with 90 mRNAs in HER2-enriched tumors in our cohort and TCGA, and they are associated with positive regulation of VEGF production. It has been documented that in tumors with overexpressed HER2 the expression and secretion of VEGF is induced, and expression increases when tumors become resistant to treatment^33^. Additionally, negative regulation of anoikis is also an enriched pathway. Anoikis is a hallmark of metastatic cells, and it has been reported that HER2 cells are resistant to this process^34^. None of the up-regulated lncRNAs have been described in HER2-enriched BRCA.

Co-expression analysis of lncRNA–mRNA highlights important functions of specific lncRNAs for each molecular subtype. Some groups of subtype-specific altered lncRNAs are involved in common pathways (RNA polymerase transcription, histone acetylation), while others are more specialized (in the HER2 subtype, those related to VEGF production), explaining the high heterogeneity of the molecular biology of breast tumors. This suggests that the up-regulation of lncRNAs may regulate processes associated with carcinogenesis, which makes them interesting targets for further studies to understand the biology of the molecular subtypes of BRCA.

The HER2-enriched molecular subtype is characterized by the amplification of genes contained in the 17q12 amplification, as well as, the high expression rate of ERBB2 gen and other five cancer promoting genes (*GRB7*, *PNMT*, *STARD3*, *PGAP3*, and *MGC1483*)^35^. HER2-enriched tumors are also one of the subtypes exhibiting the lowest progression free survival^36^ and the highest rate of metastasis and lower OS in the Mexican cohort. Therefore, biological characterization of HER2-enriched tumors is a relevant task, especially for deciphering the role of genes located within the HER2 amplification zone.

Few reports have focused on the study of lncRNAs in the HER2-enriched molecular subtype^37^. To date, no one has investigated the functional role of the AC009283.1, a lncRNA up-regulated in HER2-enriched subtype and localized in an amplified region that has been documented to be necessary for the carcinogenic process in this subtype. To better understand the function of the AC009283.1 in the regulation of global gene expression, we examined the effect of its knockdown on the SKBR3 cell transcriptome. To the best of our knowledge, AC009283.1 alterations and biological roles have not previously been reported in breast cancer, thus this would be the first study to describe them.

Microarray analysis revealed that AC009283.1 knockdown regulates 158 genes, some associated with proliferation, cell cycle and apoptosis, as shown by enrichment analysis pathway. This analysis found that AC009283.1 knockdown altered the expression of *NOTCH3*, *TNFa* and *FOSB*, genes that have been previously suggested to be drivers for proliferation and cell cycle in cancer. Up-regulation of *NOTCH3* induces cell cycle arrest at the G0/G1–S phase, and inhibits proliferation and colony-formation in BRCA cell lines^38^. *NOTCH3* was up-regulated by the AC009283.1 knockdown and we observed cycle cell arrest in S phase. *TNFa* gene expression was down-regulated by the knockdown of AC009283.1 and *TNFa* up-regulation has also been shown to increase cellular proliferation^39^. The oncogene FOSB was up-regulated after AC009283.1 knockdown, and *FOSB* has been associated with suppressed cell proliferation in gastric cancer cell lines^40^. In vitro assays (automated cell counting, proliferation index and cycle cell assay by flow cytometry) confirmed that knockdown of AC009283.1 reduced proliferation and decreased S phase in SKBR3 cells, potentially through the modulation of *NOCTH3*, *TNFa* and *FOSB* genes.

Our experimental analysis showed that the expression of *BCL2A1*, *DNML1* and *KLF6*, previously reported to be associated with apoptosis, was affected by knockdown of AC009283.1. BCL2A1, an anti-apoptotic gene, is overexpressed in a variety of cancer cells, including hematological malignancies and solid tumors^41^ and *DNM1L* mediates mitochondrial and peroxisomal division and is involved in the regulation of apoptosis. In a previous work, the knockdown of DNM1L expression caused significant increase in apoptosis^42^. Both of these genes were down-regulated after AC009283.1 knockdown. Finally KLF6 is a tumor suppressor that is down-regulated or mutated in several types of cancers, suppressing tumor growth through the activation of p21 and inducing apoptosis in prostate cancer^43^. Knockdown of AC009283.1 causes up-regulation of *KLF6*. Thus, increased apoptosis in SKBR3 cells with knockdown of AC009283.1, may be through the modulation of BCL2A1, DNML1 and KLF6 genes.

Pathway enrichment analysis from microarray analyses and our in vitro assays demonstrated that AC009283.1 knockdown has an anti-proliferative effect and enhances apoptosis in the SKBR3 cell line. Thus, overexpression of AC009283.1 in breast tumors induces apoptosis evasion and sustained proliferation, both processes being hallmarks of cancer^44^. The dual regulation of proliferation and apoptosis that we observe in this work seems to be common for cancer lncRNAs. One example is LSINCT5 that regulates 36 protein-coding genes in breast and ovarian cancer^45^, GAS5 in lung cancer^46^, and HOXA11-AS in gastric cancer through regulation of miR-1297^47^ and UCA1 in colorectal cancer^48^. In this context, our results suggest that AC009283.1 contributes to the malignant phenotype in the HER2-enriched subtype through the interaction with key genes, leading to increased proliferation and resistance to death by apoptosis.

To further validate the functional biological role of AC009283.1 in HER2-enriched tumors driven by HER2 amplification, we performed a GSEA and ssGSEA on two independent breast cancer cohorts. GSEA analysis shows that over-expression of AC009283.1 is associated with over-representation of cellular proliferation and cell cycle progression enrichment pathways. This data was confirmed by ssGSEA. Moreover, cellular processes are driven by multiple molecules that interact with each other. Knowing that genes that participate in the same signaling and that share similar functions will tend to co-express^49^, Cemitool co-expression analysis was performed. We found modules significantly enriched in HER2+ tumors overexpressing AC009283.1 related to cell proliferation and metabolism. Some immunological process were also over-represented. Since the HER2 subtype has been described as an immunogenic tumor, a detailed study of this phenotype and the establishment of its relation with AC009283.1 should be included in future studies^50^.

Data resulting from diverse computational strategies suggest that AC009283.1 is a potential driver for the malignant phenotype in HER2 enriched/amplified tumors, confirming our results from the in-vitro knockdown assays. The molecular mechanism by which AC009283.1 regulates the expression of target genes involved in proliferation, cell cycle and apoptosis remains unknown. Previous studies have reported that a large part of lncRNAs regulate transcription through chromatin interaction and modulation^9^. We found the AC009283.1 is enriched in the cell nucleus so it is likely that its functions take place in the chromatin. Therefore, further studies are needed in order to understand the molecular mechanism of AC009283.1 in HER2-enriched BRCA.

## Conclusions

We found that the expression of a significant number of lncRNAs is deregulated in BRCA. High expression of AC009283.1 may be causally associated with carcinogenesis and we suggest that it plays a potential role in HER2-enriched breast cancer. Additional studies to clarify the regulatory mechanisms of lncRNAs in BRCA will improve our understanding of their contribution to the tumor phenotype.

## Methods

### Breast tissue collection

Human BRCA specimens (n = 74) (Luminal A = 24, Luminal B = 23, HER2 = 14, Basal = 13) and adjacent breast tissue (n = 12) were obtained from the Institute of Breast Diseases, FUCAM, between 2008–2015. None of the patients received neoadjuvant therapy. The adjacent tissues were collected 2 cm from the tumor margin. All tissues were frozen in liquid nitrogen and stored at − 80 °C until further use. Histological evaluation by two trained pathologists confirmed diagnosis and only samples with more than 80% of tumor cells were included in the analysis. This study was approved by the Research and Ethics Committee of National Institute of Genomic Medicine and the Institute of Breast Diseases, FUCAM (CE2009/11). Research was performed in accordance with relevant guidelines and regulations. Written informed consent was obtained from each patient before any procedure.

Overall survival (OS) of the BRCA TCGA and METABRIC patients was analyzed with the multivariable Cox regression model. Relative Risk was also calculated. This analysis was performed using the PASW statistics software (SPSS, IBM). Disease-free survival (DFS) of our Mexican cohort was analyzed with the Kaplan–Meier model. For all statistical tests, the level of significance was < 0.05.

### Cell lines

Human BRCA cell lines included in the analyses (MCF7, ZR-75-1, MDA-MB-361, SKBR3, MDA-MB-468, HCC1187, HS578T, MDA-MB-231, MDA-MB-453) and non-tumorigenic epithelial cell line MCF10A, were purchased from American Type Culture Collection (ATCC, Manassas, VA, USA). Cell lines were grown according to ATCC guidelines, supplemented with 10% fetal bovine serum (FBS) (ATCC) in a humidified incubator at 37 °C with 5% CO_2_.

### RNA extraction

Frozen tissues were disrupted using a TissueRuptor (Qiagen Inc, Valencia, CA) and RNA extraction was performed with the DNA-RNA AllPrep System (Qiagen Inc, Valencia, CA). RNA from cell lines was extracted by Trizol (Invitrogen). RNA concentration was evaluated by spectrophotometry (NanoDrop Technologies, Wilmington, Delaware), and RNA integrity was analyzed using the BioAnalyzer 2100 (Agilent Technologies, Palo Alto, CA). Only samples with a RNA integrity number (RIN) above 8 were used for microarray analysis. Total RNA was stored at − 80 °C until processing.

### Quantitative reverse transcription PCR (RT-qPCR)

cDNA was synthetized using SuperScript III RT-PCR (Invitrogen) following the manufacturer’s recommendations. Briefly, 100 nanograms (ng) of total RNA from cell lines were used to synthesize cDNA in a final reaction volume of 20 μL. The PCR mixture contained 1 μL of cDNA, 5 μL 2 × TaqMan Universal Master Mix (Applied Biosystems, Cat 4304437), 0.5 μL TaqMan probes (Hs_01590788 custom from AC009283.1) and 3.5 μL of nuclease-free water. GAPDH (Hs99999905) and SCARNA (Hs03391742_cn) were used as endogenous controls.

### Microarray expression

Human Transcriptome Array 2.0 (Affymetrix, Inc, Santa Clara, CA) was used to measure the mRNA and lncRNA expression following the manufacturer’s recommendations. 200 ng of total RNA were processed with the WT Plus Reagent Kit protocol and Affymetrix hybridization kit.

### Gene expression profiles

Transcriptome data from Affymetrix HTA2 data was analyzed using Affymetrix’s Expression and Transcriptome Analysis Consoles. According to the manufacturer, the array platform contains 44,699 protein-coding genes and 22,829 non-protein coding genes, also called Transcript Cluster ID (TCID), however, only 62% of all TCID (41,572) is annotated, the remaining 38% of TCID (25,956) is unannotated. In order to have a high-quality reference gene annotation, the TCIDs in the Affymetrix array were re-annotated with the BioMart-Ensembl tool. In this manner, we identified 10,153 lncRNAs that were included in the microarray HTA 2.0 (Supplementary data 1.4).

Gene expression data from our Mexican cohort was processed to determine BRCA molecular subtypes according to the PAM50 expression profile^3^. Initially, tumor samples were compared to normal adjacent tissues, and secondly each molecular subtype was compared to the other subtypes, using an unpaired one-way ANOVA test. Transcripts were considered to be differentially expressed when fold change was > 2.0, ANOVA p-value < 0.05, and false discovery rate (FDR) < 0.05.

To validate our findings regarding the highly expressed lncRNAs in breast tumors from the Mexican cases, we used TCGA level 3 data and selected lncRNAs with potential clinical significance in breast tumors through TANRIC. In the TCGA cohort, for the validation of lncRNA expression in tumors relative to normal adjective tissue, we used RNAseq data from The Cancer lncRNome Atlas (TCLA)^51^. Expression data from The Cancer Genome Atlas in the Atlas of Non-Coding RNA (TANRIC) (https://ibl.mdanderson.org/tanric/_design/basic/download.html) was used as an independent validation dataset for the differential expression of lncRNAs across molecular subtypes.

### Co-expression analysis

To identify co-expressed lncRNA–mRNA pairs, Pearson correlation coefficients were calculated based on the expression value between every differentially expressed lncRNA and mRNA pair using LncSubpathway for the Mexico data and the TANRIC tool for the TCGA data. The threshold of Pearson correlation coefficients was set to > 0.3 from TCGA data and > 0.4 from Mexico data, and the corresponding p-value was set to < 0.05. The Gene ontology (GO) analyses for co-expression sets were completed using the ClueGO app in Cytoscape, using mRNA that correlates with lncRNAs in both cohorts.

### Breast cancer TCGA data sets for HER2 analysis

The HT-Seq raw counts from gene expression RNAseq were downloaded from Xena data base (https://xenabrowser.net/datapages/). Data was annotated with biomaRt^52^^,^ Gencode v33 (https://www.gencodegenes.org/human/) and LNCipedia^53^. All transcripts annotated as processed transcript, lncRNA, lincRNA, antisense, nc RNA, ncRNA intronic, sense intronic, sense overlapping and prime overlapping ncrna were considered as lncRNAs. Segmentation file from Affymetrix Genome-Wide Human SNP Array 6.0 was also retrieved from the Xena data base (copy number segments—after removing germline cnv). Additionally, clinical information was downloaded. HER2+ tumors, based on immunochemistry (IHC) evaluations, validated when available with CISH HER2 information, were selected for further analysis. GEO data sets processed with the Affymetrix Human Genome U133 Plus 2.0 Array included in this study: GSE20711, GSE29431 ,GSE5460, GSE17907, GSE29044, and GSE66305 only HER2+ tumors by IHC.

### RNA-seq expression profiles

Low expressed (< 10 counts) raw count data were filtered. Then, normalization and differential expression profiles were computed using DESeq2 on DESeq2 package on R^53^^,^ independently analyzing coding and lncRNA transcripts.

### Generation of somatic copy number alteration profiles

To determine significantly recurrent regions on segmented copy number data from HER2+ (IHC) tumors, GISTIC (Genomic Identification of Significant Targets in Cancer) 2.0 algorithm^54,55^ was computed on a Genepattern server (https://cloud.genepattern.org/gp/pages/index.jsf) with the following parameters: deletion threshold = 0.1, cap values = 1.5, broad length cutoff = 0.7, remove X-Chromosome = 0, confidence Level = 0.90, Join Segment Size = 4, arm level Peel Off = 1, Maximum sample segments = 2000 and Gene GISTIC = 1.

### Neighboring coding and noncoding transcripts within the 17q-21 amplicon

Genes contained (core genes) in each significant identified wide peak (q < 0.05) on 17q-21 amplicon by GISTIC analysis, were determined by Genome browser table tool, using Gencode v32. Then, a set of lncRNA–mRNA and mRNA–mRNA pair neighboring core genes were assembled using the GenomicRanges package^56^. Two categories were defined based on the proximity to the core genes: (1) close gene: the nearest neighbor genes and (2) distant genes: the nearest neighbor gene of the defined close genes. Genes were considered if they were expressed in at least 50% of the evaluated samples.

### Co-expression module identification and building of consensus network

HER2+ tumors with different AC009283.1 expression levels were classified as: low expressing tumors (AC009283.1 levels < 1st quantile), medium (> 1st quantile and < 3rd quantile) and high (> 3rd quantile). DeSeq2 normalized counts of coding genes and AC009283.1, belonging to the above described classes were submitted to CEMiTool^57^ on R environment, using default parameters (Pearson correlation coefficient and unbiased selection of genes using a variance-based filter). The Pearson correlation coefficient between each pair of genes in the evaluated tumor expression profiles was computed. Genes from each module were connected through a network. Genes significantly correlated (Pearson > 30%, p < 0.05) with AC009283.1 were annotated in each network. CEMiTool GSEA was carried out by using the curated gene sets^58^ (C1-Hallmark gene sets, C2-Kegg curated genes, and C5GO-BP gene sets) of the Molecular Signature Database (MSigDB) (https://www.broad.mit.edu/gsea/).

### Implementation of GSEA and ssGSEA

A non-preranked GSEA^59^ was performed by applying the C1-Hallmark gene sets^58^^,^ C2-Kegg curated genes and C5GO-BP gene sets of the MSigDB using the R package GSVA^60^. FDR < 0.25 is considered to be statistically significant for GSEA. Individual scores for each tumor sample were computed by the ssGSEA method^61^ implemented in GSVA R package using the following parameters: min gene set size of 5 and normalization. The evaluated gene sets were: proliferation UNC337 intrinsic clustering, hallmark apoptosis, GO regulation of cell cycle g2 m phase transition, GO negative regulation of cell cycle arrest and GO cell cycle g2 m phase transition. ssGSEA scores were then plotted by ggplot library. DeSeq2 normalized RNA-Seq data from coding genes were employed to compute GSEA and ssGSEA scores.

### Subcellular fractionation

Cellular fractionation (cell nucleus and cytoplasm) was performed using Ambion PARIS Kit (Thermo Fisher, Cat AM1921) according to manufacturer’s instructions. RNA was isolated and qRT-PCR was performed as described above.

### AC009283.1 knockdown

Short hairping RNAs (shRNAs) specifically targeting AC009283.1 were designed using BLOCK-iT U6 RNAi Entry Vector Kit (Invitrogen, Cat K494500) following the manufacturer’s instructions. We generated the double-stranded oligo (dsoligo) and subsequently performed the ligation reaction of ds oligo into the pENTR /U6 vector, which was used to transform competent *E. coli* One Shot TOP10. We used Sanger sequencing to corroborate the presence and correct orientation of the dsoligo on the vector.

Two designed shRNA were used to knockdown AC009283.1 (shRNA1 and shRNA2). shRNA1: top 5′CACCGAGGAAGTAGGTTAGGGAATGCGAACATTCCCTAACCTACTTCCTC3′, bottom 5′AAAAGAGGAAGTAGGTTAGGGAATGTTCGCATTCCCTAACCTACTTCCTC3′ and shRNA2: top 5′CACCGCAAATAGGTGTCTCATAGCTCGAAAGCTATGAGACACCTATTTGC3′ and bottom 5′AAAAGCAAATAGGTGTCTCATAGCTTTCGAGCTATGAGACACCTATTTGC3′. negative control (NC): top: 5′CACCGGAATTACGGAGTCTTCTTCGCGAACGAAGAAGACTCCGTAATTCC3′ and bottom: 5′ AAAAGGAATTACGGAGTCTTCTTCGTTCGCGAAGAAGACTCCGTAATTCC3′ with a sequence that does not target mRNAs in the human genome (Green Fluorescence Protein, GFP).

Knockdown of AC009283.1 in HER2-enriched cell line (SKBR3 ATCC HTB-30) was accomplished using two shRNAs (shRNA-1 and shRNA-2), which were transfected using Xfect Transfection Reagent Protocol-At-A-Glance (Clontech, Palo Alto, CA, Cat 631318). Briefly, 3.5 × 10^5^ SKBR3 cells were seeded in 6-well plates and 3 μg of plasmid were transfected during 48 h. Nanoparticle complexes were removed and replaced with complete growth medium McCoy5A (ATCC). 96 h post-transfection, expression of AC009283.1 was evaluated by RT-qPCR. The RNA was extracted by TRIzol (Thermo Scientific, Cat 15596026) and stored at − 80 °C. Independent experiments were performed in triplicate.

The effect of AC009283.1 knockdown on the transcriptional landscape of SKBR3 cell was analyzed with the HTA 2.0 microarray (Affymetrix, Santa Clara, USA) as described above, we performed three microarrays for shRNA NC and three for shRNA 2 (AC009283.1 knockdown). Genes with a fold change of > 1.2 and < − 1.2, p-value < 0.05 were considered as significant and selected for biological pathway analysis using Ingenuity Pathway Analysis (IPA). To explore the effect of the AC009283.1 in tumor samples, we performed a differential expression analysis of HER2-enriched tumor samples with high and low expression of AC009283.1 (first and fourth quantile). Count tables of RNASeq were obtained from TCGA data portal and subsequently differential expression was performed with DESeq2. We obtained a list of differentially expressed genes and used it for biological pathway analysis with IPA.

### Cellular proliferation assay

Cell proliferation of AC009283.1 knockdown cells was analyzed using two methods: (1) counting the total number of positive cells stained with trypan blue in a TC20 Automated Cell Counter (BIO-RAD), and (2) detecting proliferation rate using Cell Trace CFSE Cell Proliferation Kit (Invitrogen, Cat C34554), according to the manufacturer’s protocol, using flow cytometry (Attune, Applied Biosystems). 7.7 × 10^4^ SKBR3 cells were seeded in 24-well plates and were transfected with shRNA or NC shRNA. Cell proliferation assays were carried out at 24, 48, 72 and 96 h. At least three independent experiments were performed for each assay.

### Apoptosis assay

7.7 × 10^4^ SKBR3 cells were seeded in 24-well plates, and incubated with shRNA for 96 h. Cells were harvested by trypsinization and washed with PBS, cells were treated with QBS 20 µM for 24 h to perform the apoptosis assay. Then, the cells were re-suspended in binding buffer and stained with Annexin V and PI (FITC Annexin V/Dead cell Apoptosis kit, Invitrogen, Carlsbad, CA, USA) for 15 min in the dark at room temperature. The stained cells were examined by flow cytometry (Attune, Applied Biosystems). The cells were categorized into early and late apoptotic cells. At least three independent experiments were performed. We conducted a second validation test of apoptosis using a caspase-3 activity assay, performed following the manufacturer’s instructions (Merck #235419). We used QBS 50 µM as positive control of apoptosis and FlowJo T v10.0 software for analysis.

### Statistical analysis

Statistical significance was estimated using GraphPadPrism (version 6, San Diego, USA). ANOVA and Student's *t* test were performed for all comparisons involving categorical variables. Correlation between variables was determined by Pearson’s correlation coefficient. The Kaplan–Meier method was assessed by log**‐**rank test and COX. *p* value < 0.05 was considered as significant (*p < 0.05, **p < 0.005, ***p < 0.0005).

### Ethical approval and informed consent

This study was approved by the Research and Ethics Committee of the National Institute of Genomic Medicine and the Institute of Breast Diseases, FUCAM (CE2009/11). Written informed consent was obtained from each patient before any procedure.

## Supplementary information

Supplementary Figures.

Supplementary Information.

Supplementary Data 1.

Supplementary Data 2.

Supplementary Data 3.

Supplementary Data 4.

Supplementary Table.

## Data Availability

To review GEO accession GSE134254: Go to https://www.ncbi.nlm.nih.gov/geo/query/acc.cgi?acc=GSE134254. Enter token enopkmqerzmvzut into the box. To review GEO accession GSE134359: Go to https://www.ncbi.nlm.nih.gov/geo/query/acc.cgi?acc=GSE134359 Enter token ehofieiuhpkbdkp into the box.
