# Supplementary material for: A lncRNA landscape in breast cancer reveals a potential role for AC009283.1 in proliferation and apoptosis in HER2-enriched subtype
Source: Sci Rep. 2020 Aug 4;10:13146. doi: 10.1038/s41598-020-69905-z (PMC7403317; doi:10.1038/s41598-020-69905-z)

A long non-coding RNA landscape in breast cancer reveals a potential role for linc-PPP1R1B in proliferation and apoptosis in HER2-enriched molecular subtype

Cedro-Tanda Alberto[1,3], Ríos Magdalena[1,6], Romero-Córdoba Sandra[1,5], Cisneros Villanueva Mireya[1], Rebollar-Vega Rosa Gloria[4], Alfaro-Ruiz Luis Alberto[1], Jiménez-Morales Silvia[1], Domínguez-Reyes Carlos[2], Villegas-Carlos Felipe[2], Tenorio-Torres Alberto[2], Bautista-Piña Veronica[2], Beltrán-Anaya Fredy Omar[1,3], Hidalgo-Miranda Alfredo[1]*

Supplementary Figure 1. Clinical outcomes across molecular subtypes in breast cancer A. Metastasis-free survival in Mexico cohort, B. Overall survival in METABRIC cohort, C. Overall survival in TCGA cohort D. Cox Proportional Hazards ratio in METABRIC and TCGA cohorts. Univariate Cox Regression was performed using Luminal A subtype as reference.

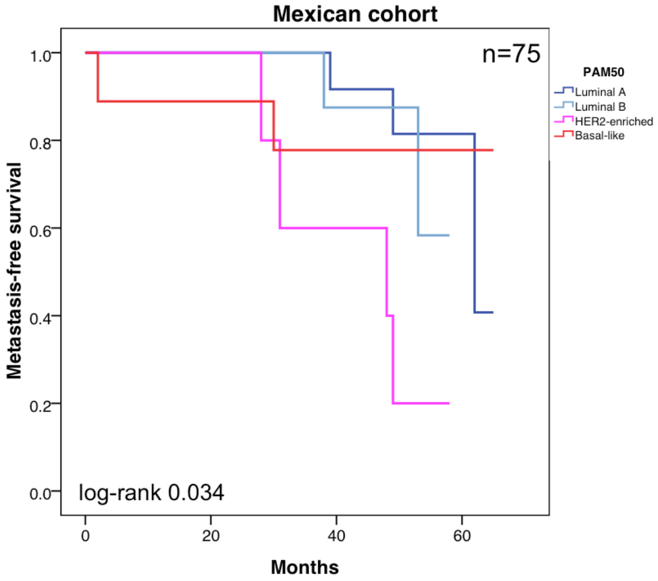

A

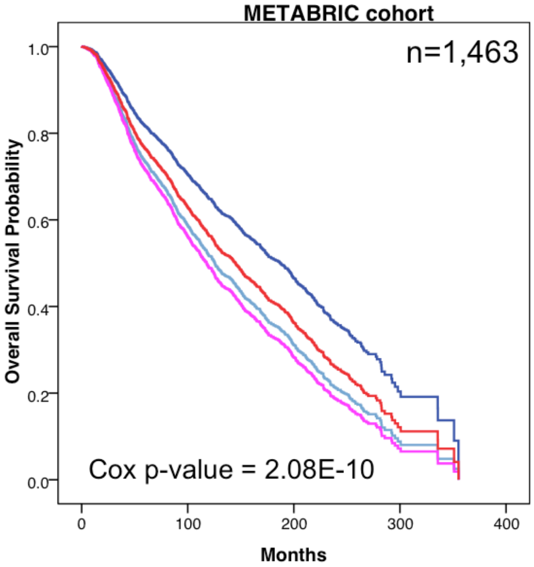

B

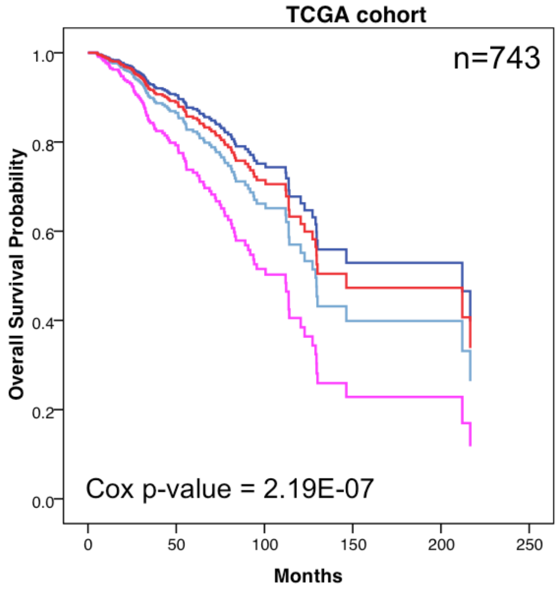

C

D

Hazard ratio	METABRIC	TCGA
Luminal B	1.445	1.529
HER2-enriched	2.320	1.655
Basal-like	1.176	1.329

Supplementary Figure 2. Overall survival for LINC01087 and RP11-379F12.4 in Luminal B tumors.

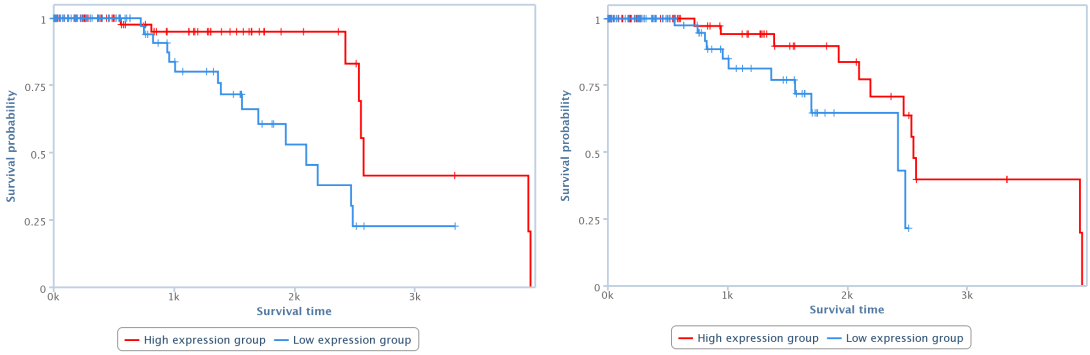

LINC01987 Cox P-value = 0.03739214

RP11-379F12.4 log-rank = 0.02798734

Supplementary Figure 3. Overall survival for U62317.2 in Basal-like tumors.

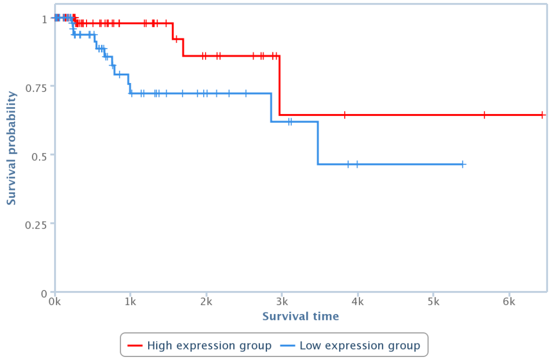

U62317.2 log-rank = 0.04378998

Supplementary Figure 4. Real time qPCR identifying SK-BR-3 cell line as a potential biological model for over-expressed AC009283.1.

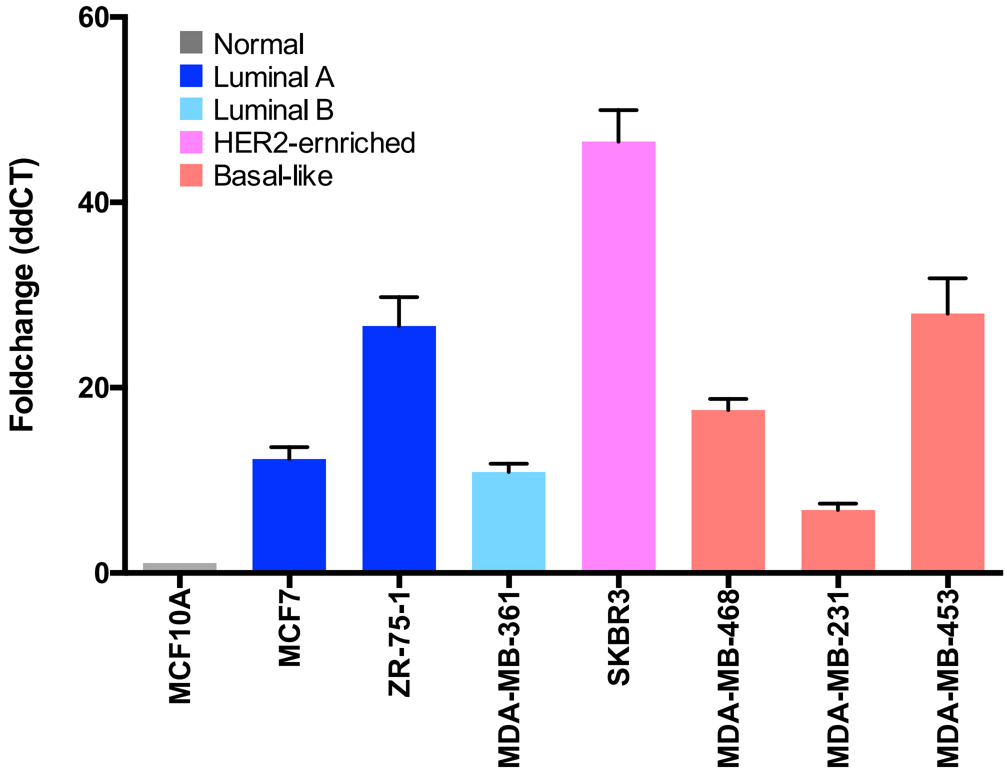

Supplementary Figure 5. Relative expression of AC009283.1 in nucleus and cytoplasm.

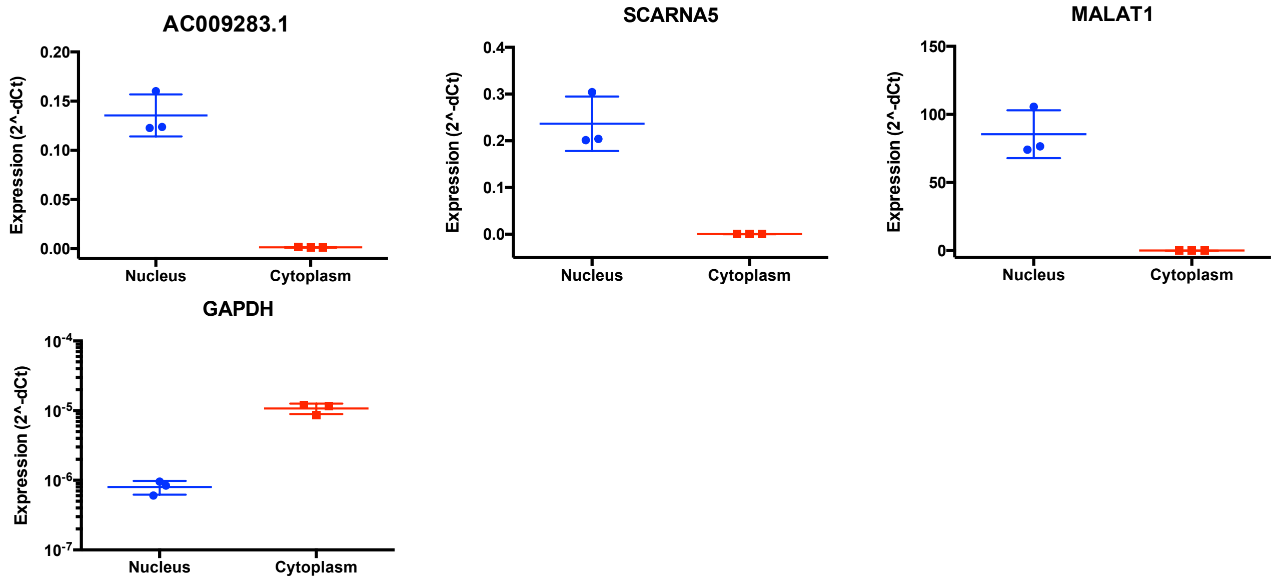

Supplementary Figure 6. Enrichment analysis of differentially expressed genes after AC009283.1 knockdown in SKBR3 cell line. Data were analyzed through the use of IPA (QIAGEN Inc., https://www.qiagenbioinformatics.com/products/ingenuitypathway-analysis).

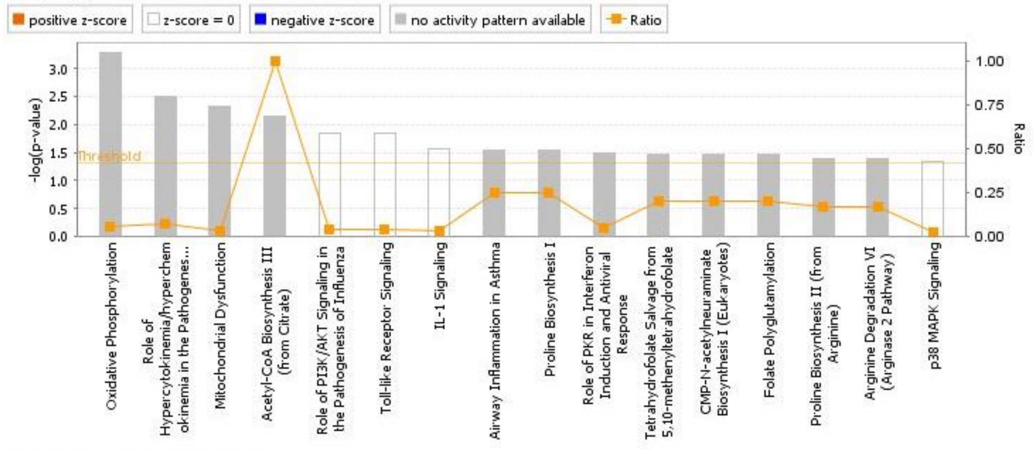

© 2000-2016 QIAGEN. All rights reserved.

Supplementary Figure 7. Correlation plots (expression vs amplification status) in top 5 up-regulated lncRNAs in the 17q12 region from HER2 tumors and its amplification status using GISTIC (loss, no change, gain and amplification).

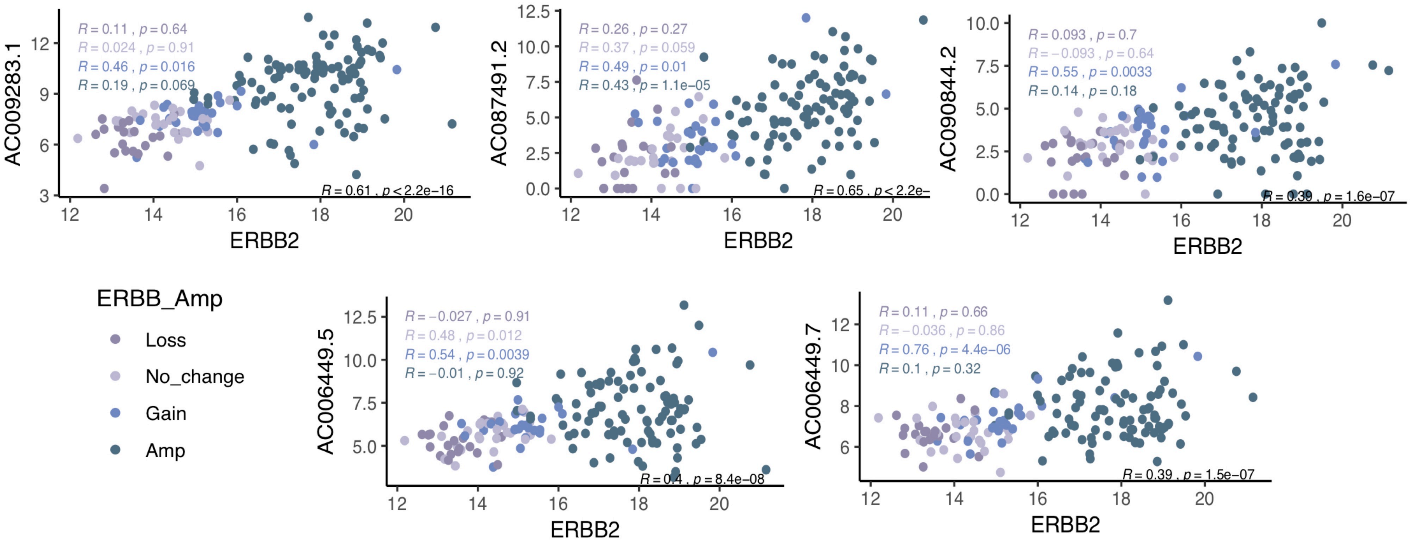

Supplement: Supplementary file 1 — Supplementary Figures. [file 41598_2020_69905_MOESM1_ESM.pdf]
