# Supplementary material for: A lncRNA landscape in breast cancer reveals a potential role for AC009283.1 in proliferation and apoptosis in HER2-enriched subtype
Source: Sci Rep. 2020 Aug 4;10:13146. doi: 10.1038/s41598-020-69905-z (PMC7403317; doi:10.1038/s41598-020-69905-z)
Supplement: Supplementary file 2 — Supplementary Information. [file 41598_2020_69905_MOESM2_ESM.docx]

**Supplementary table 1.docx** Clinical and pathological features.

**Supplementary data 1.xlsx**

1.1 307 lncRNAs differentially expressed betwwen tumor breast tissues from normal adjacent. Foldchange >2, -<2. FDR <0.05.

1.2 Differentially expressed gene (coding and non-coding) betwwen tumor breast tissues from normal adjacent. Without filters.

1.3 lncRNAs up and down regulated (tumor vs normal adjacent tissue) in mexican cohort and TCGA cohort.

1.4 TCID were anotated with BIOMART tool by ENSEMBL.

**Supplementary data 2.xlsx**

**2.1** Expression profile of lncRNAs in breast cancer subtypes from mexican cohort. Microarray data. Foldchange >1.5, <-1.5. FDR <0.05. Each molecular subtype was compared to the others.

**2.2** Expression profile of lncRNAs in breast cancer subtypes from TCGA cohort. RNA-Seq data. Log foldchange >1, <-1. adj P-value <0.05. Each molecular subtype was compared to the others.

**Supplementary data 3.xlsx**

**3.1.** Co-expression patterns of four lncRNAs RNAs up-regulated with mRNAs in Luminal A. Coefficient is correlation Pearson.

**3.2.** Co-expression patterns of four lncRNAs RNAs up-regulated with mRNAs in Luminal B. Coefficient is correlation Pearson.

**3.3.** Co-expression patterns of three lncRNAs RNAs up-regulated with mRNAs in Basal-like. Coefficient is correlation Pearson.

**3.4.** Co-expression patterns of three lncRNAs RNAs up-regulated with mRNAs in HER2-enriched. Coefficient is correlation Pearson

**Supplementary data 4.xlsx**

**4.1.** Differentially expressed genes in SKBR3 with shRNA 2 AC009283.1 vs shRNA NC

**4.2.** Differentially expressed genes in HER2-enriched tumors (TCGA data) with high expression VS low expression of AC009283.1.

**Supplementary figures.docx**

**Figure 1.** Clinical outcomes across molecular subtypes in breast cáncer **A**. Metastasis-free survival in Mexico cohort, **B.** Overall survival in METABRIC cohort, **C.** Overall survival in TCGA cohort **D.** Cox Proportional Hazards ratio in METABRIC and TCGA cohorts. Univariate cox Regression was performed using Luminal A subtype as reference.

**Figure 2.** Overall survival for LINC01087 and RP11-379F12.4 in Luminal B tumors

**Figure 3.** Overall survival for U62317.2 in Basal-like tumors.

**Figure 4.** Real time qPCR identifying SK-BR-3 cell line as a potential biological model for up-expressed AC009283.1

**Figure 5.** Relative expression of AC009283.1in nucleus and cytoplasm.

**Figure 6.** Enrichement analysis of diferentially expressed genes after AC009283.1 knockdown in SKBR3 cell line. Was performed with Ingenuity Pathways Analysis (IPA).
