# Supplementary material for: A lncRNA landscape in breast cancer reveals a potential role for AC009283.1 in proliferation and apoptosis in HER2-enriched subtype
Source: Sci Rep. 2020 Aug 4;10:13146. doi: 10.1038/s41598-020-69905-z (PMC7403317; doi:10.1038/s41598-020-69905-z)
Supplement: Supplementary file 7 — Supplementary Table. [file 41598_2020_69905_MOESM7_ESM.docx]

| **Characteristics** | **n = 75** | **100%** |
| --- | --- | --- |
| **Age (years)**  <40  40-60  60-80  >80  Unknown |  | |
|  | 4 | 5.3% |
|  | 36 | 75% |
|  | 20 | 26.7% |
|  | 3 | 4% |
|  | 12 | 16% |
| **Hormonal receptors (IHC)**  **ER**  Positive  Negative  Unknown |  | |
|  | 50 | 66.7% |
|  | 20 | 26.7% |
|  | 5 | 6.7% |
| **PR**  Positive  Negative  Unknown |  | |
|  | 37 | 49.3% |
|  | 33 | 44% |
|  | 5 | 6.7% |
| **HER2**  Positive  Negative  Unknown |  | |
|  | 12 | 16% |
|  | 55 | 73.3% |
|  | 8 | 10.7% |
| **Molecular subtype (PAM50)**  Luminal A  Luminal B  HER2-enriched  Basal-like |  | |
|  | 24 | 32% |
|  | 24 | 32% |
|  | 14 | 19% |
|  | 13 | 17% |
| [**Breast Cancer Stages:**](https://www.breastcancer.org/symptoms/diagnosis/staging)  0  I  II  III  IV  Unknown |  | |
|  | 0 | 0% |
|  | 5 | 6.7% |
|  | 50 | 66.7% |
|  | 6 | 8% |
|  | 1 | 1.3% |
|  | 13 | 17.3% |
| **Tumor grade**  G1  G2  G3  G4  Unknown |  | |
|  | 3 | 4% |
|  | 37 | 49.3% |
|  | 14 | 18.7% |
|  | 0 | 0% |
|  | 21 | 28% |
| **Histopathological Diagnosis**  Invasive ductal carcinoma  Others  Unknown |  | |
|  | 56 | 74.7% |
|  | 6 | 8% |
|  | 13 | 17.3% |
| **Overall Survival**  <5 years  >5 years  Unknown |  | |
|  | 52 | 69.3% |
|  | 5 | 6.7% |
|  | 18 | 24% |
| **Metastasis**  Yes  No  Unknown |  | |
|  | 11 | 14.7% |
|  | 37 | 49.3% |
|  | 27 | 36% |
